# The new-generation selective ROS1/NTRK inhibitor DS-6051b overcomes crizotinib resistant ROS1-G2032R mutation in preclinical models

**DOI:** 10.1038/s41467-019-11496-z

**Published:** 2019-08-09

**Authors:** Ryohei Katayama, Bo Gong, Noriko Togashi, Masaya Miyamoto, Masaki Kiga, Shiho Iwasaki, Yasuki Kamai, Yuichi Tominaga, Yasuyuki Takeda, Yoshiko Kagoshima, Yuki Shimizu, Yosuke Seto, Tomoko Oh-hara, Sumie Koike, Naoki Nakao, Hiroyuki Hanzawa, Kengo Watanabe, Satoshi Yoda, Noriko Yanagitani, Aaron N. Hata, Alice T. Shaw, Makoto Nishio, Naoya Fujita, Takeshi Isoyama

**Affiliations:** 1 Division of Experimental Chemotherapy, Cancer Chemotherapy Center, Japanese Foundation for Cancer Research, Tokyo, 135-8550 Japan; 2 Department of Medical Genome Science, Graduate School of Frontier Science, The University of Tokyo, Tokyo, 108-8639 Japan; 3 Daiichi Sankyo Co., Ltd, Tokyo, 140-8710 Japan; 4 Daiichi Sankyo RD Novare Co., Ltd, Tokyo, 134-8630 Japan; 5 Massachusetts General Hospital Cancer Center, Boston, MA 02129 USA; 6 Department of Medicine, Harvard Medical School, Boston, MA 02115 USA; 7 Department of Thoracic Medical Oncology, the Cancer Institute Hospital, Japanese Foundation for Cancer Research, Tokyo, 135-8550 Japan

**Keywords:** Drug development, Non-small-cell lung cancer, Cancer therapeutic resistance

## Abstract

*ROS1* gene rearrangement was observed in around 1–2 % of NSCLC patients and in several other cancers such as cholangiocarcinoma, glioblastoma, or colorectal cancer. Crizotinib, an ALK/ROS1/MET inhibitor, is highly effective against *ROS1*-rearranged lung cancer and is used in clinic. However, crizotinib resistance is an emerging issue, and several resistance mechanisms, such as secondary kinase-domain mutations (e.g., ROS1-G2032R) have been identified in crizotinib-refractory patients. Here we characterize a new selective ROS1/NTRK inhibitor, DS-6051b, in preclinical models of ROS1- or NTRK-rearranged cancers. DS-6051b induces dramatic growth inhibition of both wild type and G2032R mutant ROS1–rearranged cancers or NTRK-rearranged cancers *in vitro* and *in vivo*. Here we report that DS-6051b is effective in treating ROS1- or NTRK-rearranged cancer in preclinical models, including crizotinib-resistant ROS1 positive cancer with secondary kinase domain mutations especially G2032R mutation which is highly resistant to crizotinib as well as lorlatinib and entrectinib, next generation ROS1 inhibitors.

## Abstract (web summary)

The treatment of ROS1-rearranged non-small cell lung cancer with the TKI crizotinib is limited due to the emergence of resistance. Here, the authors develop a new ROS1/NTRK inhibitor, DS-6051b, which overcomes crizotinib resistance in preclinical models.

## Introduction

Several genetic alterations that aberrantly activate tyrosine kinases have been identified as oncogenic drivers in non-small cell lung cancer (NSCLC), and many tyrosine kinase inhibitors (TKIs) have been developed to target these driver oncogene products^1–5^. Among the driver oncogenes, chromosomal rearrangements of anaplastic lymphoma kinase *(ALK),* ROS proto-oncogene 1 *(ROS1)*, rearranged during transfection (*RET)*, and neurotrophic receptor tyrosine kinases *(NTRKs)* are each observed in 0.1–5% of NSCLC patients^1,2,5,6^. *ROS1* gene rearrangements are observed in ~1–2% of NSCLC patients and in cholangiocarcinoma, glioblastoma, ovarian, gastric, and colorectal cancers^7^. The ROS1 oncogenic fusion protein can be targeted by the ALK/ROS1/MET TKI crizotinib^8,9^, which is approved for the treatment of metastatic ROS1-rearranged NSCLC. In a phase 1/2 clinical trial (PROFILE 1001), the overall response rate to crizotinib was 72%, and median progression-free survival reached 19.2 months^10^.

In most cases of ROS1-rearranged NSCLC, crizotinib induces marked tumor shrinkage. However, the majority of patients will develop resistance to crizotinib within a few years of treatment. Several secondary mutations in the ROS1 tyrosine kinase domain have been identified from the molecular analysis of crizotinib-refractory ROS1-rearranged NSCLC cancer patient samples^11,12^. The ROS1 G2032R mutation is analogous to the ALK G1202R mutation identified in crizotinib-, ceritinib-, and alectinib-resistant ALK-rearranged lung cancers. ROS1 G2032R is also analogous to the G595R mutation in NTRK1 identified in entrectinib-resistant NTRK1-rearranged cancer.

We previously reported that the ALK G1202R mutation confers high-level resistance to first- and second-generation ALK inhibitors examined^13^, but is sensitive to the third-generation ALK/ROS1 inhibitor lorlatinib (PF-06463922)^14^. Lorlatinib has been reported to inhibit ROS1 G2032R, but its half-maximal inhibitory concentration (IC_50_) is much higher with ROS1 G2032R compared with ALK G1202R^15^. We previously found that cabozantinib, a clinically approved multi-kinase inhibitor for thyroid and kidney cancers, is active against ROS1 G2032R in vitro, but yet to be evaluated in vivo or in clinical setting^16^. As ROS1 G2032R is the most common mechanism of resistance to crizotinib in ROS1-rearranged NSCLC^11^, the identification of therapeutic strategies to overcome ROS1 G2032R is critically important.

The *NTRK* gene family consists of *NTRK1, NTRK2,* and *NTRK3*. It encodes a receptor tyrosine kinase, and plays an important role in the nervous system in a ligand-dependent manner. However, in cancer, *NTRK* is often observed as a fusion oncogene, such as *TPM3-NTRK1*, which induces constitutive activation of NTRK1 tyrosine kinase resulting in cancer progression. Since the growth of NTRK fusion-positive cancers depends on TRK tyrosine kinase activity, TRK tyrosine kinase inhibitors induce a marked tumor growth inhibition^17^. Indeed, several TRK inhibitors, such as entrectinib or larotrectinib, have demonstrated remarkable responses in NTRK-rearranged cancers across various cancer types, including lung, colorectal, thyroid, and pediatric cancers^18,19^.

Here, we develop and characterize a novel ROS1/NTRK inhibitor, DS-6051b, which is a potent and selective ROS1 and TRK family inhibitor capable of inhibiting ROS1 G2032R and other crizotinib-resistant ROS1 mutants. We investigate the preclinical antitumor activity of DS-6051b using a variety of preclinical models, including: (1) Ba/F3 and other cell lines expressing the NTRK1 fusion or CD74-ROS1 fusion protein with or without resistant mutations, (2) ROS1-rearranged cancer patient-derived cell lines, and (3) in vivo models.

## Results

### DS-6051b Inhibits ROS1 Kinase in nanomolar concentrations

**Fig. 1:**
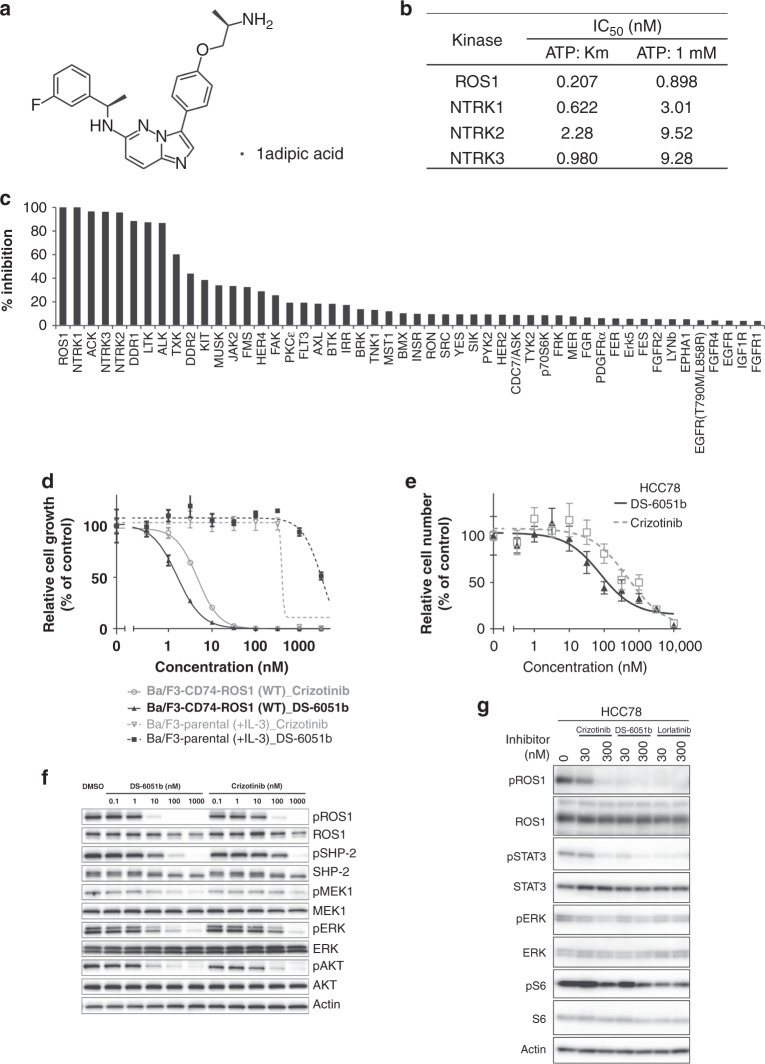
Development of the ROS1/NTRK inhibitor DS-6051b and the efficacy of DS-6051b in vitro. **a** Chemical structure of DS-6051b. **b** In vitro kinase assay in the presence of DS-6051b and the indicated recombinant ROS1 or NTRK family kinases. The calculated IC_50_ values are indicated. **c** In vitro kinase assay in the presence of 0.2 μM DS-6051b across 160 kinases at 1 mM ATP. Percentage of kinase inhibition by DS-6051b is shown in the bar graph. **d**, **e** Sensitivity of CD74-ROS1 induced or parental Ba/F3 cells (**d**) and HCC78 cells (**e**) to crizotinib or DS-6051b. The cells were treated with a range of inhibitor doses for 72 h. Parental Ba/F3 cells were treated with inhibitors in the medium containing IL-3. Cell viability was assessed using CellTiter-Glo assay. **f**, **g** Inhibition of ROS1 phosphorylation by ROS1 inhibitors in CD74-ROS1−expressing Ba/F3 cells (**f**) and HCC78 cells (**g**). Both the cell lines were treated with increasing concentrations of inhibitors for 2 h. The cell lysates were then immunoblotted to detect indicated proteins

To obtain a potent and selective ROS1 inhibitor, high-throughput inhibitor screening against ROS1 kinase was performed, and several hit compounds were identified. After robust chemical derivatization and characterization, we found a lead compound that had inhibitory activities against ROS1 and NTRKs, and lead optimization finally identified DS-6051b (Fig. 1a). DS-6051b potently inhibited recombinant ROS1, NTRK1, and NTRK3 in sub-nanomolar concentration in an ATP-competitive manner (Fig. 1b). Besides ROS1 and NTRKs, DS-6051b almost completely inhibited ACK, ALK, DDR1, and LTK at 0.2 μM among 160 kinases in the presence of 1 mM ATP, but did not inhibit other 152 kinases strongly (Fig. 1c; Supplementary Fig 1a and Supplementary Table 1a). In addition, DS-6051b inhibited NTRK2 with a few nanomolar IC_50_ values at approximately Km value for ATP, and 40 nM of DS-6051b potently inhibited several other kinases, such as NuaK1, JAK2, and MUSK, at approximately Km value of each kinase for ATP (Supplementary Table 1b). DS-6051b inhibited the growth of the CD74-ROS1 overexpressed Ba/F3 cells and the ROS1 fusion-positive HCC78 cancer cell line with a lower IC_50_ than crizotinib (Fig. 1d, e). Consistent with the cell viability assay data, DS-6051b potently inhibited autophosphorylation of ROS1 in CD74-ROS1–expressed Ba/F3 cells and the SLC34A2-ROS1 harboring HCC78 cells at approximately single-digit to double-digit nanomolar concentration (Fig. 1f, g).

### Antitumor activity of DS-6051b in ROS1-rearranged PDC models

**Fig. 2:**
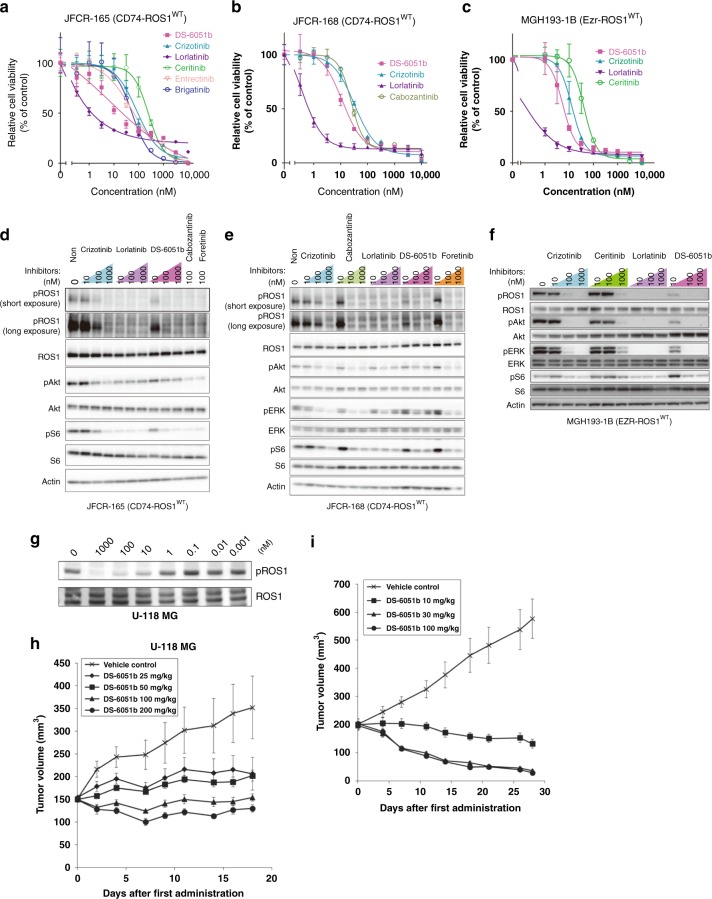
DS-6051b-inhibited patient-derived lung cancer cell lines with ROS1 fusion. **a**–**c** Sensitivity of JFCR-165 (**a**), JFCR-168 (**b**), and MGH193-1B cells (**c**) to the indicated ROS1 inhibitors, such as DS-6051b, crizotinib, lorlatinib, or cabozantinib. The cells were treated with a range of inhibitor doses for 72–120 h. Cell viability was assessed using CellTiter-Glo assay. **d**–**f** Inhibition of phosphorylation of ROS1 and downstream growth signaling-related molecules by ROS1 inhibitors in JFCR-165 (**d**), JFCR-168 (**e**), and MGH193-1B (**f**) cells. All the cell lines were treated with the indicated inhibitors at increasing concentrations for 6 h. The cell lysates were then immunoblotted to detect the indicated proteins. **g** U-118 MG cells, which is the GBM cell line harboring *FIG-ROS1* fusion gene, were treated with the increasing concentration of DS-6051b for 2 h. The cell lysates were immunoblotted to detect the phospho-ROS1. **h** U-118 MG cells were subcutaneously implanted into Balb-c *nu/nu* mice. DS-6051b (25, 50, 100, and 200 mg/kg) or vehicle was treated once daily by oral gavage for 18 days. The results in (**h**) are indicated as mean ± SE of the tumor volume of each group (*N* = 5); parametric Dunnett’s test was conducted between the DS-6051b-treated groups and the vehicle-treated control group (25 and 50 mg/kg: *P *<* 0.05, 100 mg/kg: **P *<* 0.01, 200 mg/kg: ***P *<* 0.001). **i** DS-6051b was evaluated in the PDX model bearing CD74-ROS1 fusion-positive NSCLC. DS-6051b (10, 30, and 100 mg/kg) or vehicle was treated once daily by oral gavage for 28 days. The results in (**i**) are indicated as mean ± SE of the tumor volume of each group (*N* = 10); parametric Dunnett’s test was conducted between the DS-6051b-treated groups and the vehicle-treated control group (10, 30, and 100 mg/kg: ***P < 0.001)

For further evaluation of DS-6051b efficacy, we established several ROS1-rearranged cancer cell lines from ROS1 fusion-positive lung cancer patients and tested the efficacy. Since no CD74-ROS1 rearrangement-harboring cancer cell line was publicly available, two CD74-ROS1 and one EZR-ROS1-rearranged cancer cells were established from the pleural effusions of three TKI-naive patients (JFCR-165, JFCR-168, and MGH-193-1). These patient-derived cells were sensitive to DS-6051b, crizotinib, lorlatinib, cabozantinib, ceritinib, brigatinib, or entrectinib. Lorlatinib has the lowest, and DS-6051b the second lowest IC_50_ among the inhibitors tested (Fig. 2a–c). Consistent with the cell viability assay data, DS-6051b, crizotinib, lorlatinib, ceritinib, cabozantinib, or foretinib potently inhibited autophosphorylation of ROS1 in JFCR-165, JFCR-168, and MGH193-1B cells (Fig. 2d–f). Next, we used a glioblastoma U-118 MG cell line, harboring the *FIG-ROS1* fusion gene, and DS-6051b treatment dose dependently inhibited autophosphorylation of ROS1 in U-118-MG cells in vitro (Fig. 2g). For in vivo experiments, DS-6051b treatment effectively inhibited tumor growth at ≥25 mg/kg DS-6051b without significant body weight loss (Fig. 2h; Supplementary Fig. 5a). In addition, we evaluated the efficacy of DS-6051b using lung cancer PDX model harboring *CD74-ROS1* fusion gene in vivo, and DS-6051b treatment markedly inhibited tumor growth at ≥10 mg/kg DS-6051b without significant body weight loss (Fig. 2i; Supplementary Fig. 5b).

### DS-6051b potently inhibits NTRK-rearranged cancer in vivo

**Fig. 3:**
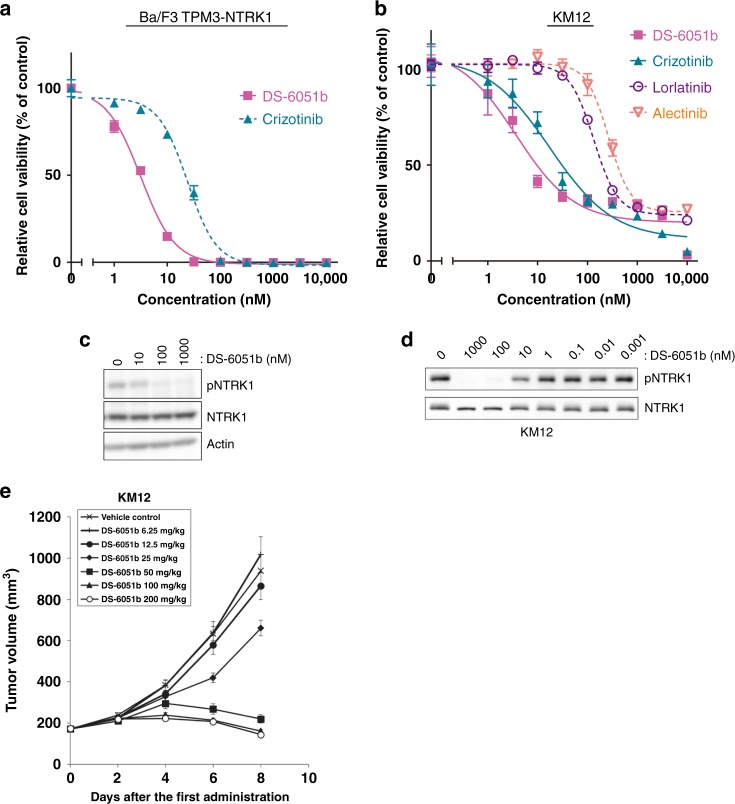
DS-6051b was active against NTRK1-rearranged cell lines. **a**, **b** Sensitivity of TPM3-NTRK1-induced Ba/F3 cells (**a**) and KM12 (**b**) to DS-6051b, crizotinib, lorlatinib, or alectinib. The cells were treated with a range of inhibitor doses for 72 h. Cell viability was assessed using CellTiter-Glo assay. **c**, **d** Inhibition of NTRK1 phosphorylation by DS-6051b in TPM3-NTRK expressing Ba/F3 cells (**c**) or KM12 (**d**) cells. Both of the cell lines were treated with increasing concentrations of inhibitors for 2 or 3 h. The cell lysates were immunoblotted to detect the indicated proteins. **e** KM12 cells were subcutaneously implanted into Balb-c *nu/nu* mice. DS-6051b (6.25, 12.5, 25, 50, 100, and 200 mg/kg) or vehicle was treated once daily by oral gavage for 8 days. The results in (**e**) are indicated as mean ± SE of the tumor volume of each group (*N* = 8); parametric Dunnett’s test was conducted between the DS-6051b-treated groups and the vehicle-treated control group (25, 50, 100, and 200 mg/kg: ***P < 0.001)

KM12 colorectal cancer cells are known to harbor the *TPM3-NTRK1* fusion gene, and are addicted to TPM3-NTRK1-mediated growth signaling. In our previous study, we developed Ba/F3 cells expressing TPM3-NTRK1 and identified multiple NTRK1 inhibitor-resistance mutations and drug candidates to overcome on-target resistance^20^. Since DS-6051b inhibited the purified NTRK family kinases at single-digit nanomolar concentration (Fig. 1b), the potency of DS-6051b using KM12 cells or BaF3-TPM3-NTRK1-expressing cells was examined. Consistently, IC_50_ of DS-6051b against Ba/F3-TPM3-NTRK1, Ba/F3-ETV6-NTRK1, -NTRK2, -NTRK3, or KM12 cells was ~3–20 nM (Fig. 3a, b; Supplementary Table 1c), and phospho-NTRK1 was partially suppressed at 10 nM, and disappeared completely by 100 nM DS-6051b treatment (Fig. 3c, d). In vivo experiments using the KM12-bearing mouse xenograft model showed that DS-6051b induced tumor shrinkage at a ≥50 mg/kg or higher treatment dose without significant body weight loss (Fig. 3e; Supplementary Fig. 5c).

### DS-6051b showed activity against NTRK1-TKI-resistant mutants

Oncogenic fusion genes involving the *NTRK* family (*NTRK1*, *NTRK2*, or *NTRK3*) have been identified in various cancers, and a few NTRK-TKIs have been effective against the NTRK-rearrangement-positive cancers in clinical trials^21,22^. Several NTRK-TKI-resistance mutations have already been identified in patients treated in those clinical trials. In addition, according to ENU mutagenesis screening, we recently identified multiple NTRK-TKI-resistant mutations using Ba/F3 models^20^. Thus, we examined the activity of DS-6051b against these resistant mutations. Consequently, the IC_50_ to the five of six NTRK1-TKI-resistant mutations, including G595R, identified in entrectinib-resistant patients was <100 nM (Supplementary Fig. 2). One mutation with G667C, also identified in entrectinib-resistant patients, was resistant to DS-6051b. Of note, G595R mutation is homologous with the known solvent front mutations ALK-G1202R and ROS1-G2032R. G667C mutation is located next to the DFG core motif.

### DS-6051b inhibits crizotinib-resistant ROS1 mutants

**Fig. 4:**
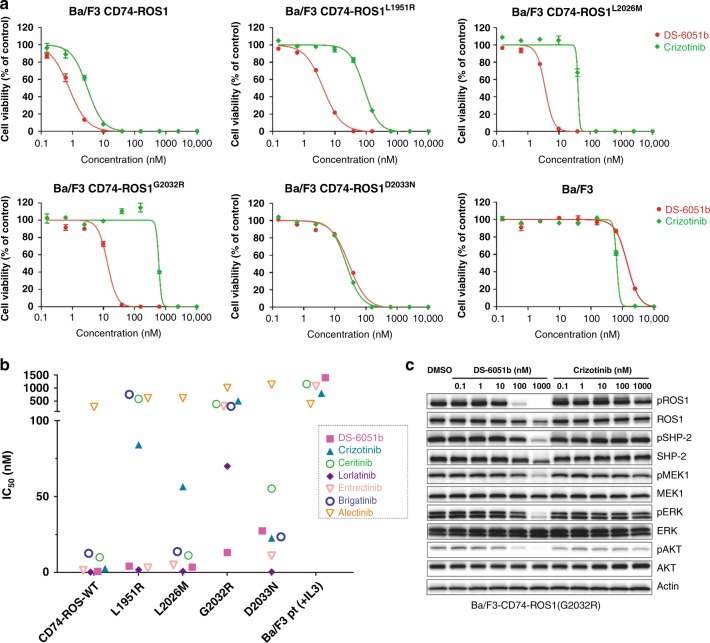
DS-6051b effectively inhibited crizotinib-resistant ROS1 secondary mutations, including G2032R solvent front mutation. **a** Sensitivity of CD74-ROS1 (WT or crizotinib-resistant mutants: L1951R, L2026M, G2032R, and D2033N) induced Ba/F3 cells or parental Ba/F3 cells to DS-6051b and crizotinib. The cells were treated with a range of inhibitor doses for 72 h. Cell viability was assessed using CellTiter-Glo assay. **b** Sensitivity of parental Ba/F3 cells (with IL-3), CD74-ROS1-wild-type, or each mutated CD74-ROS1 expressing Ba/F3 cells to various ROS1 inhibitors or ALK inhibitor. The calculated IC_50_ is shown in the graph. **c** Inhibition of phosphorylation of ROS1 and downstream growth signaling-related molecules by ROS1 inhibitors in Ba/F3-CD74-ROS1-G2032R cells. The cells were treated with increasing concentrations of DS-6051b or crizotinib for 2 h. The cell lysates were immunoblotted to detect the indicated proteins

In the previous study, we and others have identified multiple crizotinib-resistant mutations, such as L2026M gatekeeper, L1951R, S1986Y/F, G2032R, or D2033N mutations in ROS1. In particular, S1986Y/F, G2032R, and D2033N were found in crizotinib-refractory ROS1-rearranged lung cancer patients. To examine the activity against these crizotinib-resistant mutants, the activity of DS-6051b was tested using Ba/F3-CD74-ROS1 mutant models in vitro, DS-6051b effectively inhibited the growth of L1951R, L2026M, S1986F, and G2032R mutant Ba/F3 cells, but lost inhibitory activity against D2033N mutant cells (Fig. 4a, b; Supplementary Figs. 1c, 3, 4). However, D2033N was sensitive to cabozantinib, as reported previously^23^. Compared with the crizotinib, ceritinib, lorlatinib, entrectinib, or brigatinib, DS-6051b inhibited autophosphorylation of ROS1 in CD74-ROS1-G2032R mutant Ba/F3 cells, in accordance with the cell viability assay data (Fig. 4c; Supplementary Fig. 4).

**Fig. 5:**
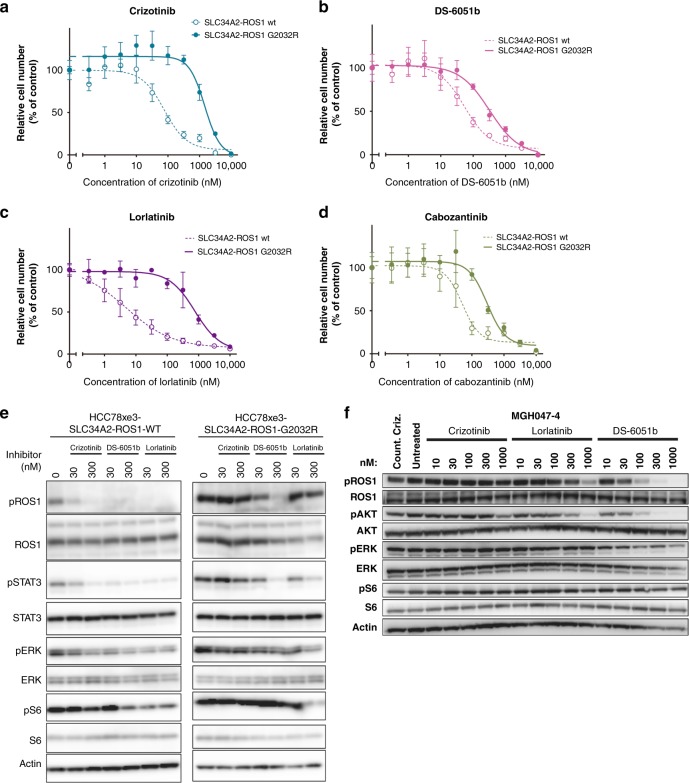
DS-6051b effectively inhibited the growth of crizotinib-resistant ROS1-G2032R mutant lung cancer cells. **a**–**d** Sensitivity of SLC34A2-ROS1 (WT or G2032R) induced HCC78xe3 cells to crizotinib (**a**), DS-6051b (**b**), lorlatinib (**c**), and cabozantinib (**d**). The cells were treated with a range of inhibitor doses for 72 h. Cell viability was assessed using CellTiter-Glo assay. **e** Inhibition of ROS1 phosphorylation by crizotinib, DS-6051b, or lorlatinib in CD74-ROS1 (WT or G2032R) induced HCC78xe3 cells. Both the cell lines were treated with increasing concentrations of the indicated inhibitors for 6 h. The cell lysates were immunoblotted to detect the indicated proteins. **f** Inhibition of phosphorylation of ROS1 and downstream growth signaling-related molecules by ROS1 inhibitors (crizotinib, lorlatinib, or DS-6051b) in MGH047-4 cells, derived from crizotinib-refractory patients harboring G2032R mutations. The cells were treated with increasing concentrations of the indicated inhibitors for 6 h. The cell lysates were immunoblotted to detect the indicated proteins

To evaluate the efficacy of DS-6051b in lung cancer cell line model especially G2032R mutant ROS1 rearranged lung cancer, attempts were made to establish the in vivo model of ROS1 fusion-positive lung cancer. Lung adenocarcinoma cell line HCC78 harbors SLC34A2-ROS1, but does not stably grow in mice with subcutaneous injection. Thus, in our recent study, we used the transplantable HCC78 sub-cell line, called HCC78xe3 cells, which stably formed tumor subcutaneously in mice by repeating to make the subcutaneous tumor in mice three times^24^. Then, we established SLC34A2-ROS1-WT and -G2032R mutant overexpressing HCC78xe3 cells. First, the sensitivity of SLC34A2-ROS1-introduced HCC78xe3 cells to crizotinib, lorlatinib, cabozantinib, or DS-6051b was tested in vitro 3D culture condition^24^. DS-6051b and cabozantinib inhibited cell growth of both WT and G2032R mutant HCC78xe3 cells at the similar concentration. In contrast, IC_50_s of crizotinib and lorlatinib to G2032R mutant cells were much higher than those to WT cells. These results suggest that DS-6051b was active against G2032R mutant lung cancer cells (Fig. 5a–e). Next, we checked whether DS-6051b can inhibit phospho-ROS1 in a patient-derived cell line. MGH047-4 cells were established from the crizotinib-refractory patient tumor harboring G2032R-mutated CD74-ROS1. As expected, DS-6051b, but not crizotinib and lorlatinib, inhibited phospho-ROS1 in MGH047-4 cells (Fig. 5f).

### DS-6051b inhibits crizotinib-resistant mutant ROS1 in vivo

**Fig. 6:**
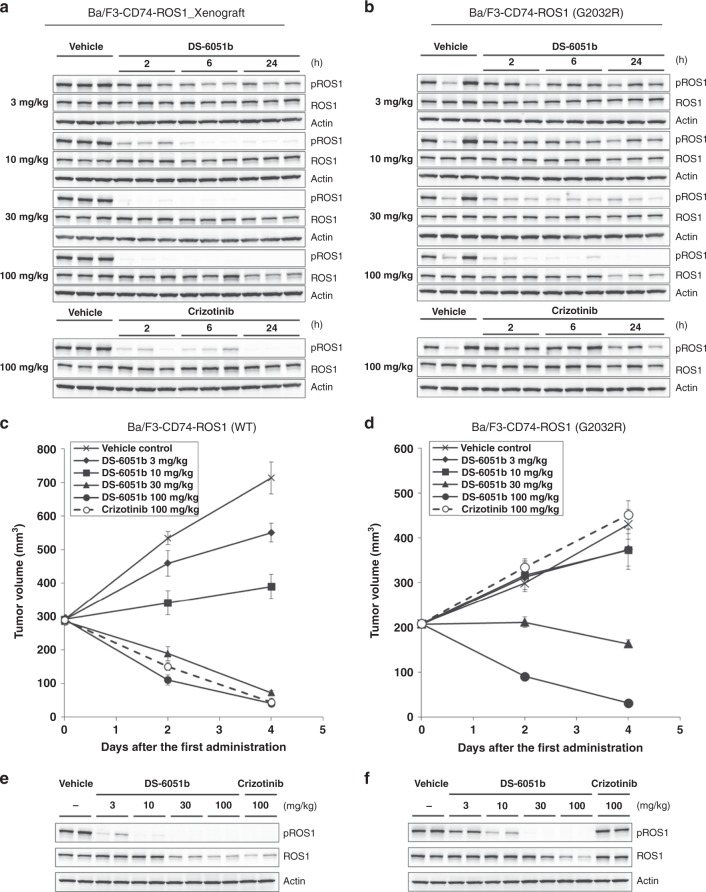
DS-6051b induced tumor regression in Ba/F3-CD74-ROS1-WT or -G2032R mutant xenograft model. **a**, **b** Mice bearing BaF3-CD74-ROS1-WT (**a**) or -G2032R (**b**) were orally administered DS-6051b at 3, 10, 30, and 100 mg/kg, or crizotinib at 100 mg/kg, and the tumors were collected and lysed at 2, 6, and 24 h post dosing. Phospho-ROS1 and ROS1 were detected by immunoblot analysis using corresponding antibodies. **c**, **d** BaF3-CD74-ROS1-WT (**c**) or -G2032R (**d**) cells were subcutaneously implanted into Balb-c *nu/nu* mice. DS-6051b (3, 10, 30, and 100 mg/kg), crizotinib (100 mg/kg), or vehicle was treated once daily by oral gavage for 4 days. The results in (**c**) and (**d**) are indicated as mean ± SE of the tumor volume of each group (*N* = 6); parametric Dunnett’s test was conducted between the DS-6051b-treated groups and the vehicle-treated control group (**c**: 3 mg/kg: **P *<* 0.01, 10, 30, and 100 mg/kg: ***P *<* 0.001; **d**: 30 and 100 mg/kg: ***P *<* 0.001). **e**, **f** Mice-bearing BaF3-CD74-ROS1-WT (**e**) or -G2032R (**f**) were orally administered DS-6051b at 3, 10, 30, and 100 mg/kg, or crizotinib at 100 mg/kg once a day for 4 days. After the fifth treatment of each drug, the tumors were collected and were lysed at 6 h post dosing. Phospho-ROS1 and ROS1 were detected by immunoblot analysis using corresponding antibodies

Since the G2032R mutation is commonly identified in crizotinib-refractory patients, the efficacy of DS-6051b in vivo using Ba/F3 cells expressing CD74-ROS1-WT or -G2032R was evaluated. These CD74-ROS1 mutants expressing Ba/F3-bearing mice were treated with various doses of DS-6051b, and tumor growth and phospho-ROS1 were inhibited in the mice xenografts in a dose-dependent manner (Fig. 6a–d). Treatment with ≥30 mg/kg of DS-6051b showed rapid tumor regression in the wild-type (WT) and the G2032R-mutant Ba/F3-bearing mice without severe body weight loss (Fig. 6c, d; Supplementary Fig. 5d, e). On the other hand, 100 mg/kg of crizotinib treatment induced tumor shrinkage in the CD74-ROS1-WT-expressing Ba/F3 xenografts only, but not in the G2032R-mutant models (Fig. 6c, d). In all, 60 mg/kg of entrectinib or 10 mg/kg of lorlatinib could not induce tumor shrinkage in G2032R mutant Ba/F3-bearing mice, although lower dose of entrectinib or lorlatinib induce complete remission in the CD74-ROS1-WT-expressing Ba/F3 xenografts (Supplementary Figs. 6a, b). In addition, single treatment of DS-6051b maintained inhibition of phosoho-ROS1 even 24 h after drug treatment (Fig. 6a, b). The Ba/F3-CD74-ROS1 (WT or G2032R) xenograft tumors treated with crizotinib or DS-6051b for 4 days were also analyzed by immunoblot. As expected, DS-6051b almost completely inhibited phospho-ROS1 of WT- and G2032R-mutated CD74-ROS1 in vivo, although crizotinib, entrectinib, or lorlatinib could completely inhibit phosoho-ROS1 in CD74-ROS1-WT-expressing Ba/F3 cells only (Fig. 6e, f; Supplementary Fig. 6d, e).

**Fig. 7:**
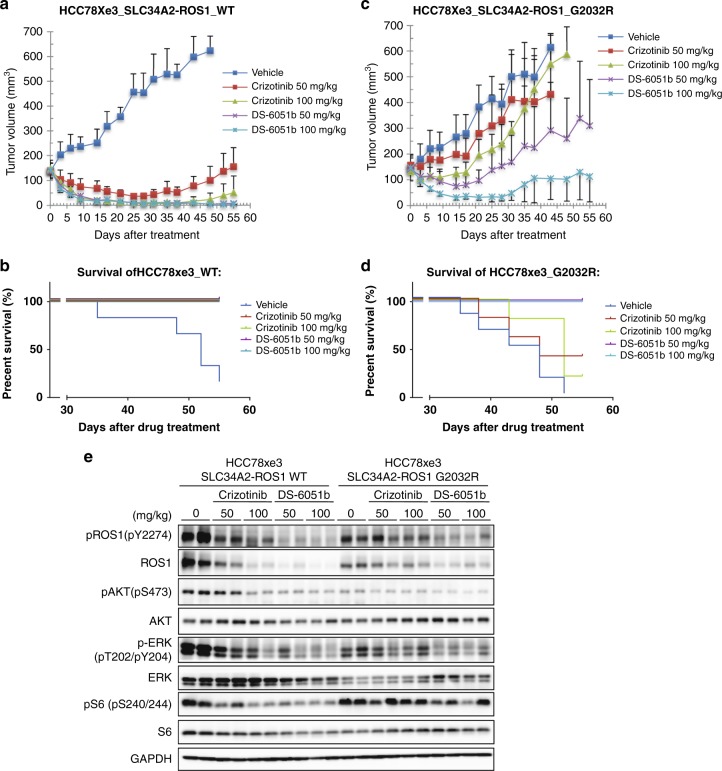
DS-6051b induced a marked tumor shrinkage to SLC34A2-ROS1-G2032R mutation harboring HCC78xe3 cells. **a**–**d** Mice-bearing SLC34A2-ROS1 (WT or G2032R) induced HCC78xe3 cells were treated with crizotinib and DS-6051b. HCC78xe3-SLC34A2-ROS1-WT (**a**) or -G2032R (**c**) cells were implanted into mice. DS-6051b (50 and 100 mg/kg) and crizotinib (50 and 100 mg/kg) were given once daily by oral gavage for the indicated period; *N* = 6. In these experiments, DS-6051b was dissolved in 0.5% MC, and crizotinib was in 0.01 N HCl. Kaplan–Meier curves of the survival of the mice in each treatment arm are shown in (**b**) and (**d**). The results in (**a**, **c**) are indicated as mean ± s.d.; **P < 0.01 (Mann–Whitney *U* test). **e** Mice-bearing SLC34A2-ROS1 (WT or G2032R) induced HCC78xe3 cells were orally administered DS-6051b at 50 and 100 mg/kg, or crizotinib at 50 and 100 mg/kg once a day for 3 days. After 3 days of each drug treatment, the tumors from two mice of each treatment groups were collected and were lysed. The indicated proteins were detected by immunoblot analysis using corresponding antibodies

Next, SLC34A2-ROS1-WT or -G2032R overexpressed HCC78xe3 cells were subcutaneously implanted into nude mice and treated with crizotinib or DS-6051b. The tumor growth itself between the WT and G2032R mutants was not significantly changed. However, as expected, the G2032R mutant HCC78xe3 xenograft tumors were highly resistant to crizotinib. On the other hand, DS-6051b treatment demonstrated significant growth inhibition in the HCC78xe3-SLC34A2-ROS1-WT xenografts at lower dose compared with crizotinib. In addition, DS-6051b inhibited G2032R mutant HCC78xe3 tumor growth without obvious toxicity and achieved prolonged survival, especially compared with the crizotinib-treated G2032R mutant xenografts which did not show any superiority to the control group (Fig. 7a–d; Supplementary Fig. 7a, b). The xenograft tumors treated with crizotinib or DS-6051b for 3 days were also analyzed by immunoblot. As expected, DS-6051b almost completely inhibited phospho-ROS1 of WT- and G2032R-mutated SLC34A2-ROS1 in vivo, although crizotinib could inhibit phospho-ROS1 in SLC34A2-ROS1-WT-expressing HCC78xe3 cells only (Fig. 7e). Since the peak plasma concentration of DS-6051b after single administration of 100 mg/kg DS-6051b was around 1500 nM (Supplementary Fig. 8), and the reported peak plasma concentrations at 600 -mg dosing in human was around 1650 nM^25^, the dosing in these in vivo experiments would be relevant. These results suggested that DS-6051b could be a potent second-generation ROS1 inhibitor against ROS1 fusion-positive cancer which are crizotinib-naive and have most crizotinib-resistant mutations.

### DS-6051b moderately inhibited ALK-TKI-resistant cells

As shown in Fig. 1c and Supplementary Table 1, DS-6051b can also inhibit ALK in vitro kinase assay with IC_50_ 32.5 nM, and inhibited growth of EML4-ALK expressing Ba/F3 cells at ~ IC_50_ 50 nM (Supplementary Fig. 9 and Supplementary Table 2). Consistent with the Ba/F3 data, DS-6051b exerted moderate inhibitory activity on the growth of ALK rearranged patients’ derived cell line models harboring various resistance mechanisms, such as secondary mutation or P-glycoprotein overexpression (Supplementary Fig. 10).

## Discussion

The identification of fusion driver oncogenes with aberrant tyrosine kinase activation, such as ALK, ROS1, RET, or NTRK1 rearrangement in cancers, is largely changing therapeutic strategies accompanied with the development of targeted therapies, especially potent TKIs. Crizotinib showed marked efficacy in phase I/II clinical trials for advanced ROS1-rearranged NSCLC, and has been approved in many countries. However, in most cases, the tumors inevitably relapse, and, to date, no treatment options with other TKIs are clinically available. In ALK-rearranged NSCLC, alectinib, ceritinib, brigatinib, and lorlatinib have demonstrated efficacy in the majority of patients after relapse on crizotinib. This was due to second-generation ALK inhibitors that were active against crizotinib-resistant mutant ALK, such as the L1196M gatekeeper or I1171T mutation. However, the G1202R solvent front mutation is highly resistant to crizotinib, alectinib, and ceritinib, and partially sensitive to brigatinib. The next-generation ALK inhibitor, lorlatinib, which is recently approved and also currently under phase III clinical evaluations, was reported to be active against G1202R-mutated ALK in preclinical and clinical studies^14,26^. Similar to ALK, multiple crizotinib-resistant mutations in ROS1 were identified in crizotinib-refractory patients or in vitro mutagenesis screening models. Among those resistant mutations in ROS1, G2032R, which is analogous to G1202R in ALK, is observed most frequently in crizotinib-refractory ROS1-rearranged NSCLC patients^7^. Although ALK-G1202R mutations can be overcome by lorlatinib, which is highly potent against ROS1, lorlatinib is less active against G2032R-mutated ROS1^24^. In our previous study, cabozantinib or foretinib was identified as being active against G2032R-mutated ROS1^16^. Currently, cabozantinib is being evaluated in clinical trials for ROS1-rearranged NSCLC. However, cabozantinib has a very high protein binding in plasma, and the majority of patients receiving cabozantinib require dose modifications due to adverse events such as gastrointestinal or skin toxicities, although the safety and tolerability profiles of cabozantinib were reported to be manageable.

In this study, we developed a ROS1 inhibitor, DS-6051b, which is active against G2032R mutant ROS1 in vitro and in vivo. In addition, DS-6051b effectively induced tumor shrinkage of the KM12 xenograft, the colorectal cancer cell with NTRK1 rearrangement. For the treatment of NTRK-rearranged cancer, multiple TRK inhibitors are under clinical evaluation, and larotrectinib and entrectinib have shown clinical efficacy in clinical trials, and larotrectinib was recently approved by US-FDA. The IC_50_ levels of those NTRK inhibitors to Ba/F3-TPM3-NTRK1 cells are almost similar^20^. Currently, DS-6051b is being evaluated in phase I clinical trials for patients with ROS1-rearranged NSCLC or ROS1- and NTRK1 fusion-positive advanced solid tumor. In the phase I clinical trial, the maximum tolerable dose was 600 mg daily with mostly grade 1 or 2 adverse events. The mean trough plasma concentrations at 400 -mg and 600 -mg dosing were ~320 ng/mL (800 nM) and 480 ng/mL (1200 nM), respectively^25^, followed by conversion to 50 nM and 75 nM as concentrations unbound to proteins. The calculated IC_50_ of G2032R-mutated CD74-ROS1 expressing Ba/F3 was 13.5 nM, which is lower than the human free concentration in plasma at 600 -mg dosing. Indeed, DS-6051b but not entrectinib or lorlatinib suppressed tumor growth of the G2032R mutant models (Ba/F3-CD74-ROS1-G2032R and HCC78xe3-SLC34A2-ROS1-G2032R) in vivo.

Besides the G2032R mutants, L1951R, S1986Y/F, L2026M, and D2033N were reported as crizotinib-resistant ROS1 mutations. DS-6051b had single-digit nanomolar IC_50_ against L1951R, S1986F, and L2026M, but against D2033N, DS-6051b had relatively high IC50 of ~30 nM. However, this D2033N already was reported to be sensitive to cabozantinib, and NSCLC patients with D2033N mutant ROS1 showed a marked response to cabozantinib^23^. In addition, we recently reported that lorlatinib is also active against D2033N mutant ROS1^24^. In a clinical trial of DS-6051b, a crizotinib-naive ROS1-rearranged NSCLC patient with brain metastasis showed a partial response in the primary lung and brain metastasized tumors, suggesting that DS-6051b would be effective in brain metastasized tumors, although the blood–brain barrier penetration of DS-6051b has not been clarified in humans yet^25^.

The limitations of this study include: (1) there is no data as to whether DS-6051b is active against G2032R mutant ROS1 in humans, (2) the other possible unidentified crizotinib-resistant mutants can be overcome by DS-6051b, and (3) not performed crystal structure analysis of DS-6051b with ROS1 kinase.

Although this study has above several limitations, the potency and effectiveness of DS-6051b against crizotinib-resistant ROS1 fusion such as G2032R, most frequently observed in clinic, and NTRK1 fusion were confirmed in multiple preclinical models in vitro and in vivo. Given that DS-6051b showed similar activities to G2032R and wild-type ROS1 fusion proteins in our models, DS-6051b expected to show the clinical efficacy even after crizotinib failure. At the time we submitted this paper, no drug had proven to overcome G2032R-mutated ROS1 fusion in clinic, but while our paper reviewed, it was reported TPX-0005 (Repotrectinib) showed activity against ROS1-G2032R mutation positive cancer patient in phase 1/2 clinical trial. Thus, if the safety and efficacy are confirmed in clinic, DS-6051b might provide therapeutic opportunities for patients with ROS1-rearranged cancer with or without crizotinib-resistant mutations, and *NTRKs*-rearranged cancers.

## Methods

### Tumor samples

Biopsy samples or pleural effusions were obtained from patients prior TKI treatment or relapsed on ROS1-TKI treatment. All patients provided informed consent for genetic and cell biological analyses, which were performed in accordance with protocols approved by the Institutional Review Board of the Japanese Foundation for Cancer Research or the Massachusetts General Hospital Cancer Center. Patient-derived cell lines are available under Cooperative Research and Development Agreement, but JFCR-165 and JFCR-168 cell lines are only available in Japan due to the approved clinical research protocol and patients’ informed consent.

### Reagents

DS-6051b, crizotinib, lorlatinib (PF-06463922), and entrectinib were synthesized at DaiichiSankyo Co. Ltd. (Tokyo, Japan). Synthetic scheme of DS-6051b was described in Supplementary Fig. 11. Cabozantinib and brigatinib (AP26113) were purchased from ShangHai Biochempartner (Shanghai, China). Alectinib and ceritinib were purchased from ActiveBiochem (Hong Kong). Each compound was dissolved in dimethyl sulfoxide (DMSO) for the cell culture experiments.

### Kinase selectivity profiling

An off-chip mobility shift assay was performed by Carna Biosciences, Inc. (Kobe, Japan), according to their product instructions. The effect of DS-6051b at a concentration of 200 nM on 160 kinases was evaluated in the presence of 1 mM ATP and expressed as % inhibition. Inhibitory activities of DS-6051b on ACK, ALK, DDR1, DDR2, KIT, LTK, ROS1, NTRK1, NTRK2, NTRK3, and TXK were determined by the mobility shift assay. ATP concentration in the assay was set at the approximate Km value of each kinase for ATP or 1 mM. The IC_50_ values were estimated according to the four-parameter logistic model.

### Isolation of genomic DNA, preparation of the total RNA and cDNA

An adequate spin column kit (Qiagen, Hilden, Germany) was used to isolate the total RNA and genomic DNA from cell pellets. The *ROS1* fusion gene or ROS1 kinase domain was amplified by PCR from the cDNA synthesized from the total RNA extracted from the HCC78 or patient-derived cells.

### Cell culture conditions

Human embryonic kidney 293FT cells (Invitrogen, Carlsbad, CA, USA) were cultured in Dulbecco’s modified Eagle medium (DMEM; Fujifilm Wako, Japan) supplemented with 10% fetal bovine serum (FBS; D-10). Ba/F3 cells, which are immortalized murine bone marrow-derived pro-B cells, were obtained from the RIKEN BRC Cell Bank (RIKEN BioResource Center), and were cultured in D-10 media with or without 0.5 ng/mL mouse interleukin (IL-3 (Invitrogen). TKI-naive or crizotinib-resistant ROS1 fusion-positive NSCLC patient-derived cells were cultured in ACL-4 medium supplemented with 3% FBS^12^. U-118 MG cells (American Type Culture Collection, Manassas, VA, USA) were cultivated with the DMEM supplemented with 10% FBS. KM12 cells (originally provided from NCI-60, National Institutes of Health, Bethesda, MD, USA) were cultured in the RPMI Medium 1640 (Thermo Fisher Scientific) supplemented with 10% FBS. HCC78 was obtained from DSMZ, and HCC78xe3 cell is a subclone of HCC78, generated by repeating subcutaneously implantation and in vitro cell culture for three times. HCC78 cells were cultured in RPMI-1640/F-12 medium with 20 mM HEPES and 7.5% FBS. All media were supplemented with 100 U/ml penicillin, and 100 µg/ml streptomycin. All cells were routinely tested and verified to be free of mycoplasma contamination. KM12 cell line was authenticated by short tandem repeat profiling analysis (BEX, Tokyo, Japan) in 2016, and all the cell lines were used within 6-month periods after thawing the original authenticated cell line stocks.

### Cell viability assays

To assess cell viability after 72-h drug treatment, 2000–5000 cells were plated in replicates of three to six in 96-well plates. Following the drug treatments, the cells were incubated with CellTiter-Glo assay reagent (Promega, Madison, WI, USA) for 10 min. A Tristar LB 941 microplate luminometer (Berthold Technologies, Oak Ridge, TN, USA) or PHERAstar Microplate Reader (BMG LABTECH GmbH, Ortenberg, Germany) was used to measure luminescence. GraphPad Prism version 7.0 (GraphPad Software, La Jolla, CA, USA) was used to graphically display the data. IC_50_ values were determined using a nonlinear regression model with a sigmoidal dose response in GraphPad.

### Immunoblot analysis

Lysates were prepared using 1x SDS lysis buffer (1% SDS and 10% glycerol in 100 mM Tris–HCl [pH 7.5])^27^. Equal amount of proteins were electrophoresed, and were immunoblotted with antibodies against phospho-ROS1 (Tyr2274, #3078), ROS1 (69D6, #3266), phospho-NTRK1 (Tyr674/675, C50F3, #4621), panNTRK (C17F1, #4609), phospho-SHP-2 (Tyr580, #5431), SHP-2 (#3397), phospho-MEK1 (Ser298, #98195), MEK1 (#12671), phospho-p42/44 ERK/MAPK (Thr202/Tyr204, #9101), p42/44 ERK/MAPK (#9102), phospho-Akt (Ser473, D9E, #4060), panAkt (C67E7, #4691), phospho-S6 ribosomal protein (Ser240/244, D68F8, #5364), S6 ribosomal protein (54D2, #2317), STAT3 (79D7, #4904), phospho-STAT3 (Tyr705, #9145) (Cell Signaling Technology, Danvers, MA, USA), GAPDH (6C5, Millipore, #CB1001), and β-actin (Sigma-Aldrich Corp., St. Louis, MO, USA). All the primary antibody was used 1:1000 dilution for immunoblot analysis.

### Retroviral infection

cDNA encoding WT or mutant CD74-ROS1, SLC34A2-ROS1-S, TPM3-NTRK1, and EML4-ALK were cloned into 1520 retroviral expression vectors (pLenti) or pLenti6.3 (Invitrogen) using LR clonase, and viruses were replicated in 293FT cells by transfecting with packaging plasmids. After retroviral infection, Ba/F3 or HCC78 cells were selected by incubation with puromycin (0.7 μg/mL) or blastcidin (7 μg/mL) for 2 weeks. For Ba/F3 cells infected by CD74-ROS1 variants, IL-3 was withdrawn from the culture medium at least 2 weeks before starting the experiments.

### Antitumor efficacy test

Specific pathogen-free female nude mice were purchased from Charles River Laboratories Japan, Inc. (Yokohama, Japan). The in vitro cultured cells were transplanted subcutaneously into female nude mice (U-118 MG suspended in Matrigel, KM12: 5 × 10^6^ cells/mouse, *CD74-ROS1* fusion gene-driven Ba/F3: 1 × 10^7^ cells/mouse, HCC78xe3 cells: 3 × 10^6^ cells/mouse). When the average estimated tumor volume was around >100 mm^3^, the mice were grouped randomly based on the estimated tumor volume. The tumor length and width in millimeters were measured using a digital caliper. The estimated tumor volume of each mouse was calculated according to the following equation:

Estimated tumor volume (mm^3^) = 1/2 × (tumor length) × (tumor width)^2^

DS-6051b and crizotinib were dissolved in 0.5% (wt/vol) methyl cellulose 400 cP solution (abbreviated as 0.5% MC; Wako Pure Chemical Industries, Ltd., Osaka, Japan) as a salt-free dehydrated form. The 0.5% MC solution was used as the vehicle. The administration volume in milliliters was calculated as 10 mL/kg of body weight. The mouse body weight was measured with a digital balance for animals. DS-6051b, crizotinib, and vehicle were administered orally once per day. Mean tumor volume and standard error or standard deviation of each group were calculated. The above animal experimental procedures were performed according to the in-house guideline of the Institutional Animal Care and Use Committee of JFCR or Daiichi Sankyo Co., Ltd.

Antitumor efficacy test on patient-derived xenograft CTG-0848, representing human NSCLC harboring the *CD74-ROS1* fusion gene, also was conducted by Champions Oncology, Inc. (Baltimore, MD, USA) according to their standard procedures.

Estimated tumor volume of each mouse on the day after the final administration of DS-6051b was used for statistical analyses. A parametric Dunnett’s test or Mann–Whitney *U* test was conducted between the DS-6051b-treated and vehicle-treated control groups to evaluate the antitumor activity of DS-6051b. For the immunoblot study with xenograft tumor, the implanted tumors were resected from the mice after drug treatment and were examined by immunoblot.

### Statistical analysis

All in vitro data are presented as means ± SD. Statistical analysis was performed using parametric Dunnett’s test or Mann–Whitney *U* test as indicated in the figure legends. Significance was established for *P-*values < 0.05.

### Reporting summary

Further information on research design is available in the Nature Research Reporting Summary linked to this article.

## Supplementary information

Supplementary Information

Description of Additional Supplementary Files

Supplementary Data 1

Supplementary Data 2

Reporting Summary

Source Data of In Vivo Experiments

Source Data of Uncropped Immunoblots

## Data Availability

The original data of Fig. 1a and Supplementary Fig. 1a are presented in the Supplementary Data 1. NGS analysis data of PDC and PDX models are presented in the Supplementary Data 2, and NGS data are deposited at National Bioscience Database Center with the accession ID: hum0194. The uncropped scans of the most important blots and tumor volumes for in vivo experiments are shown as Source Data. The X-ray crystallographic coordinate for structure reported in this study has been deposited at the Cambridge Crystallographic Data Centre (CCDC), under deposition number 1910921. All the other data supporting the findings of this study are available within the article and its Supplementary Information files and from the corresponding author upon reasonable request. A reporting summary for this article is available as a Supplementary Information file.
