# Supplementary material for: The new-generation selective ROS1/NTRK inhibitor DS-6051b overcomes crizotinib resistant ROS1-G2032R mutation in preclinical models
Source: Nat Commun. 2019 Aug 9;10:3604. doi: 10.1038/s41467-019-11496-z (PMC6688997; doi:10.1038/s41467-019-11496-z)
Supplement: Supplementary file 1 — Supplementary Information [file 41467_2019_11496_MOESM1_ESM.pdf]

The new-generation selective ROS1/NTRK inhibitor DS-6051b overcomes crizotinib resistant ROS1-G2032R mutation in preclinical models

Katayama R. et al

Supplementary Figure 1

a

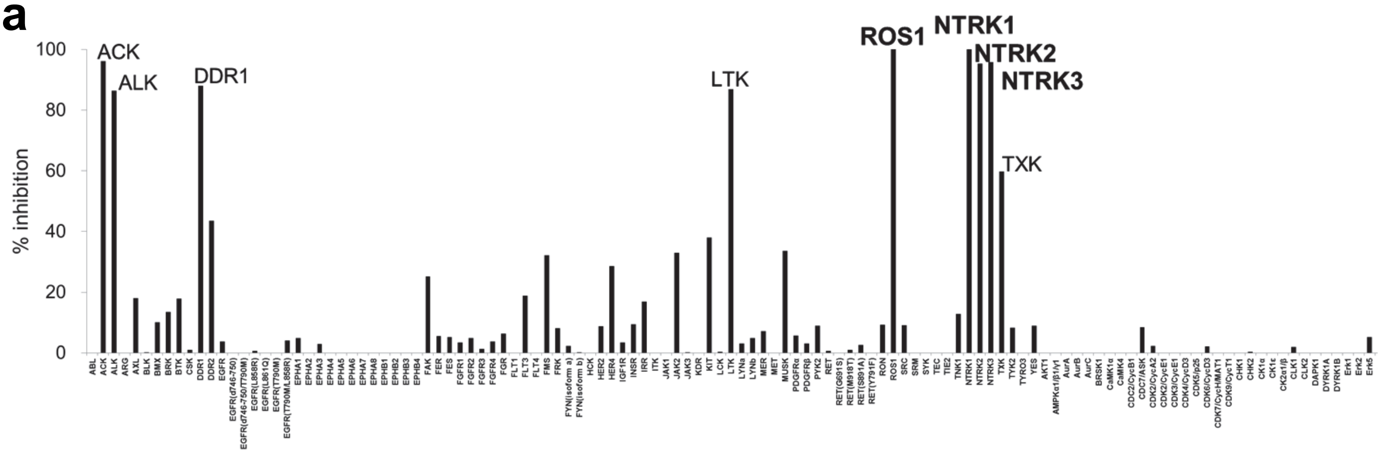

b

Cell Line	IC$_{50}$ (nM)						
	DS-6051b	Crizotinib	Ceritinib	Lorlatinib	Entrectinib	Brigatinib	Alectinib
Ba/F3-CD74-ROS1	0.8	3.0	11.2	<0.2	1.7	13.8	302.6
Ba/F3-CD74-ROS1^{L1951R}	4.2	86.1	598.0	1.8	3.0	766.7	616.5
Ba/F3-CD74-ROS1^{L2026M}	3.6	56.5	11.3	0.8	5.0	13.9	630.9
Ba/F3-CD74-ROS1^{G2032R}	13.5	525.2	405.7	73.6	321.1	309.1	1062.2
Ba/F3-CD74-ROS1^{D2033N}	28.3	23.2	56.3	0.4	11.2	24.2	1173.3
Ba/F3	1414.9	812.4	906.1	9959.3	1082.3	1848.6	396.1

c

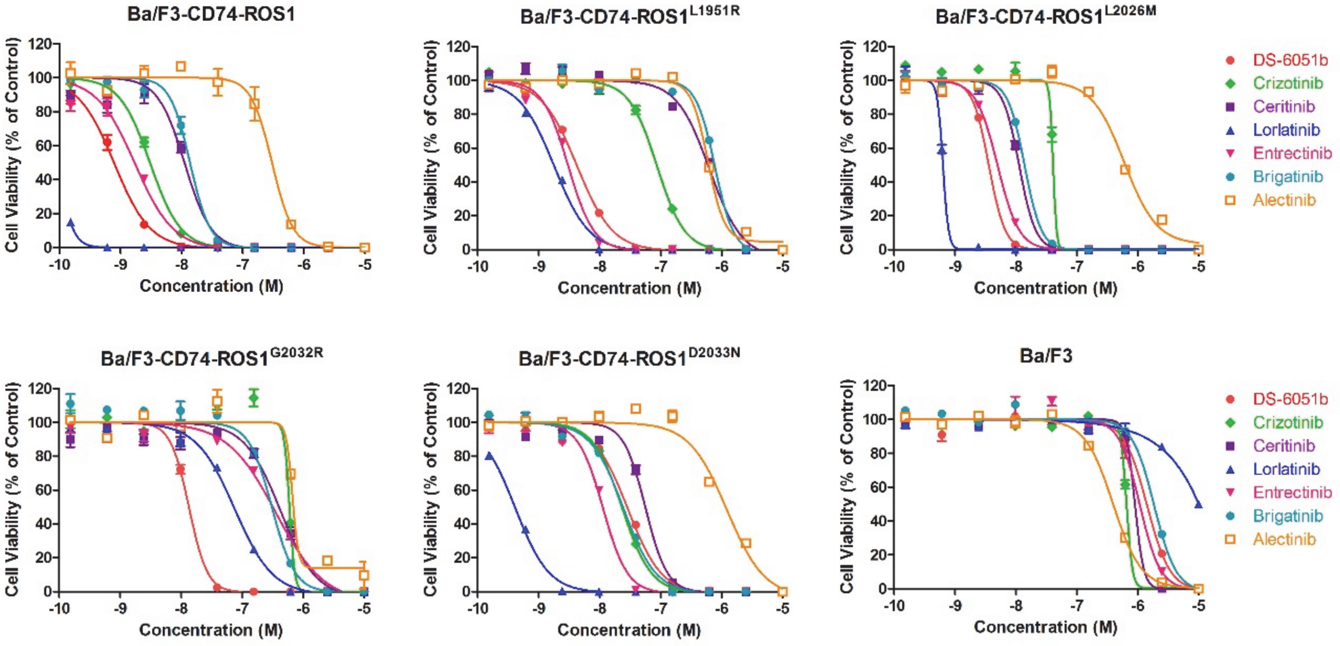

Supplementary Figure 1. Selectivity and efficacy of the novel ROS1/NTRK inhibitor DS-6051b
(a) *In vitro* kinase assay in the presence of 0.2 μM DS-6051b across 160 kinases. % of kinase inhibition by DS-6051b is shown in the bar graph. (b, c) Sensitivity of CD74-ROS1 (WT and crizotinib resistant mutants) introduced or parental Ba/F3 cells to DS-6051b, Crizotinib, Ceritinib, Lorlatinib, Entrectinib, Brigatinib, or Alectinib. The cells were treated with a range of inhibitor doses for 72 h. Parental Ba/F3 cells were treated with inhibitors in the medium containing IL-3. Cell viability was assessed using CellTiter-Glo assay. The calculated IC50 data were shown in (b) and the representative data were shown in (c).

Supplementary Figure 2

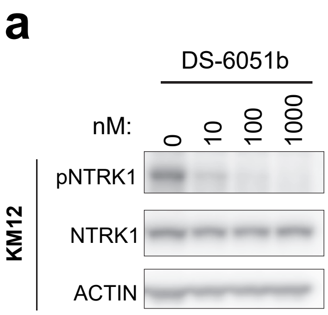

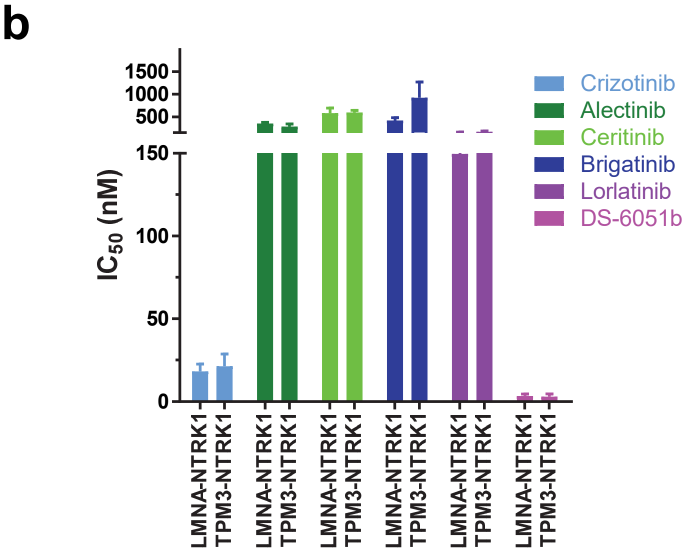

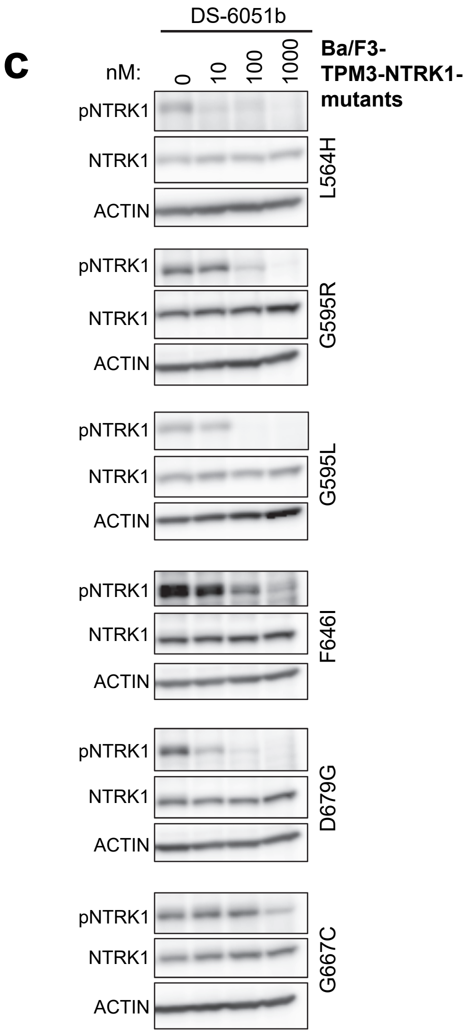

d

Ba/F3 TPM3-NTRK1 mutants

Ba/F3 line	IC_{50} (nM) to DS-6051b
WT	1.88
L564H	25.8
G595R	85.6
G595L	29.2
F646I	25.6
D679G	1.8
G667C	304.1
parental (+IL3)	>1000

Supplementary Figure 2. DS-6051b was active against NTRK1-rearranged cancers

(a, c) Inhibition of NTRK1 phosphorylation by DS-6051b in KM12 cells (a) or TPM3-NTRK1 (WT or mutants) expressing Ba/F3 cells (c).These cells were treated with increasing concentrations of DS-6051b, and the cell lysates were then immunoblotted to detect indicated proteins. (b) Sensitivity of LMNA-NTRK1 or TPM3-NTRK1–induced Ba/F3 cells to DS-6051b. The cells were treated with a range of inhibitors doses for 72 h. Cell viability was assessed using CellTiter-Glo assay. The calculated IC50 was shown as bargraph.

(d) Sensitivity of TPM3-NTRK1 (WT and mutants)–induced or parental Ba/F3 cells to DS-6051b. The cells were treated with a range of inhibitor doses for 72 h. Cell viability was assessed using CellTiter-Glo assay. The calculated IC50 was shown in table.

Supplementary Figure 3

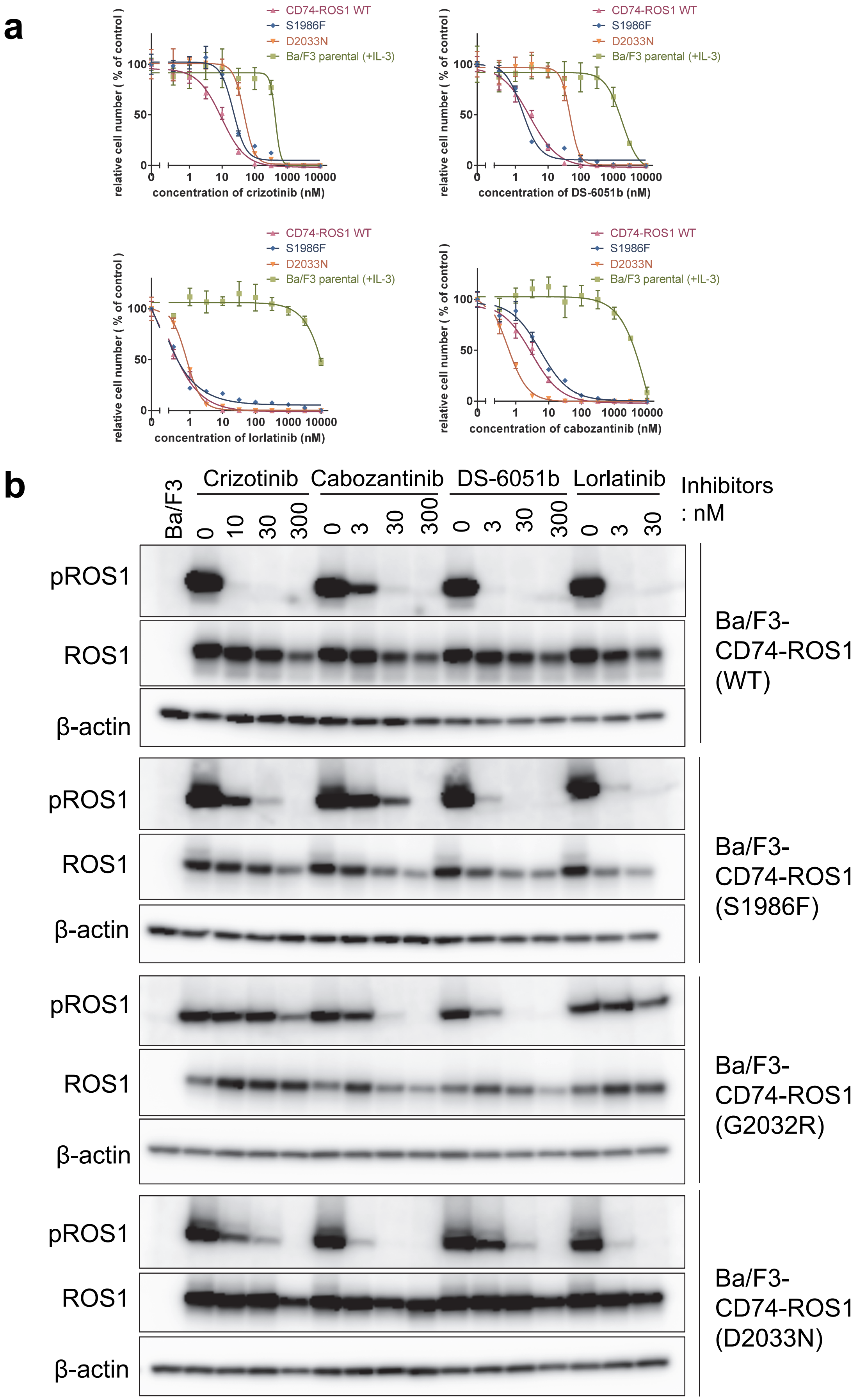

Supplementary Figure 3. DS-6051b was active against most of the crizotinib resistant mutations.
(a) Sensitivity of CD74-ROS1 (WT and crizotinib resistant mutants) introduced or parental Ba/F3 cells to DS-6051b, Crizotinib, Lorlatinib, or cabozantinib. The cells were treated with a range of inhibitor doses for 72 h. Parental Ba/F3 cells were treated with inhibitors in the medium containing IL-3. Cell viability was assessed using CellTiter-Glo assay. (b) The above Ba/F3 cells were treated with increasing concentrations of inhibitors, and the cell lysates were then immunoblotted to detect indicated proteins.

Supplementary Figure 4

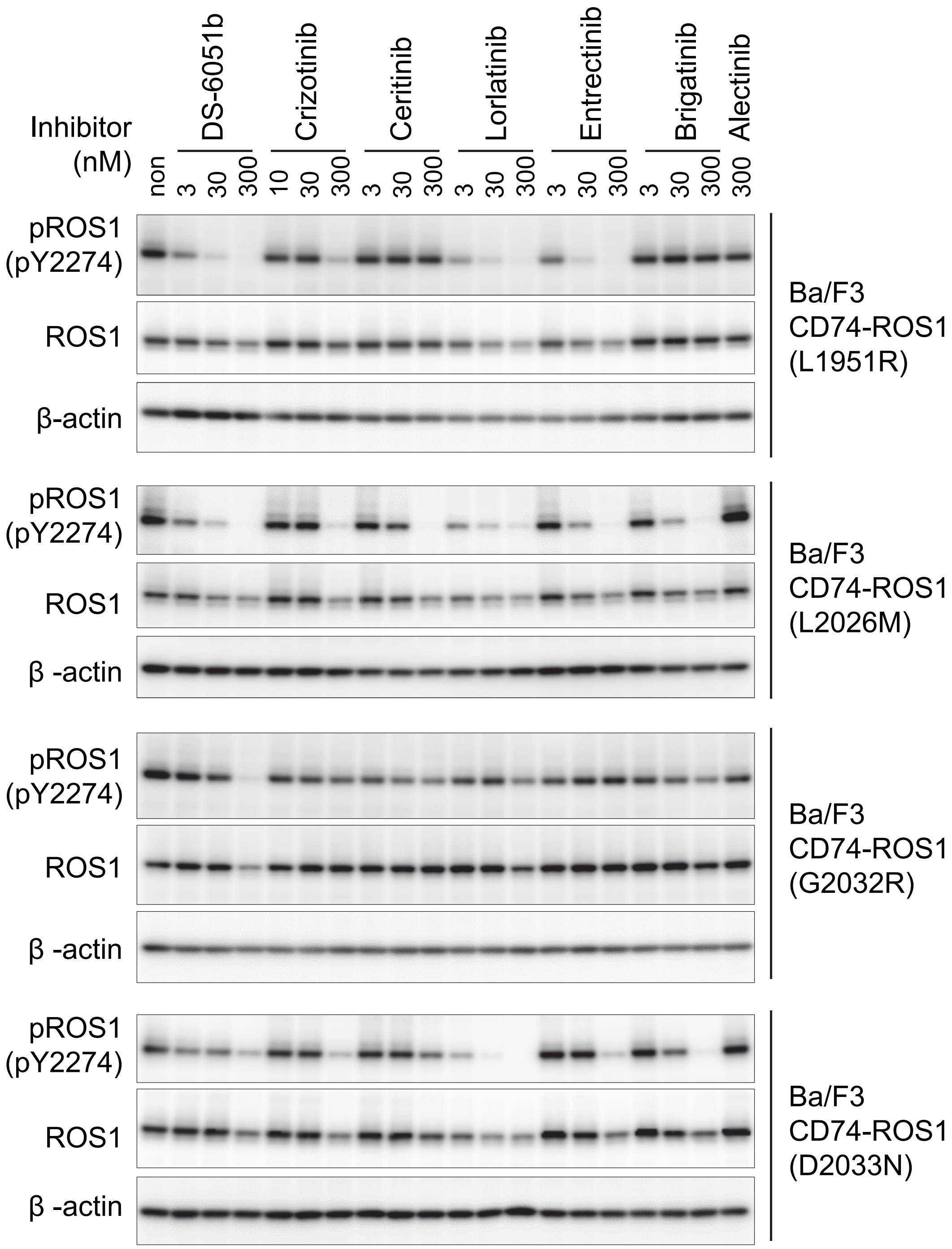

Supplementary Figure 4. ROS1 inhibitor sensitivity of ROS1 resistant mutants

Sensitivity of CD74-ROS1 (WT and crizotinib resistant mutants) introduced or parental Ba/F3 cells to DS-6051b, Crizotinib, Lorlatinib, or cabozantinib. The cells were treated with increasing concentrations of inhibitors, and the cell lysates were then immunoblotted to detect indicated proteins.

Supplementary Figure 5

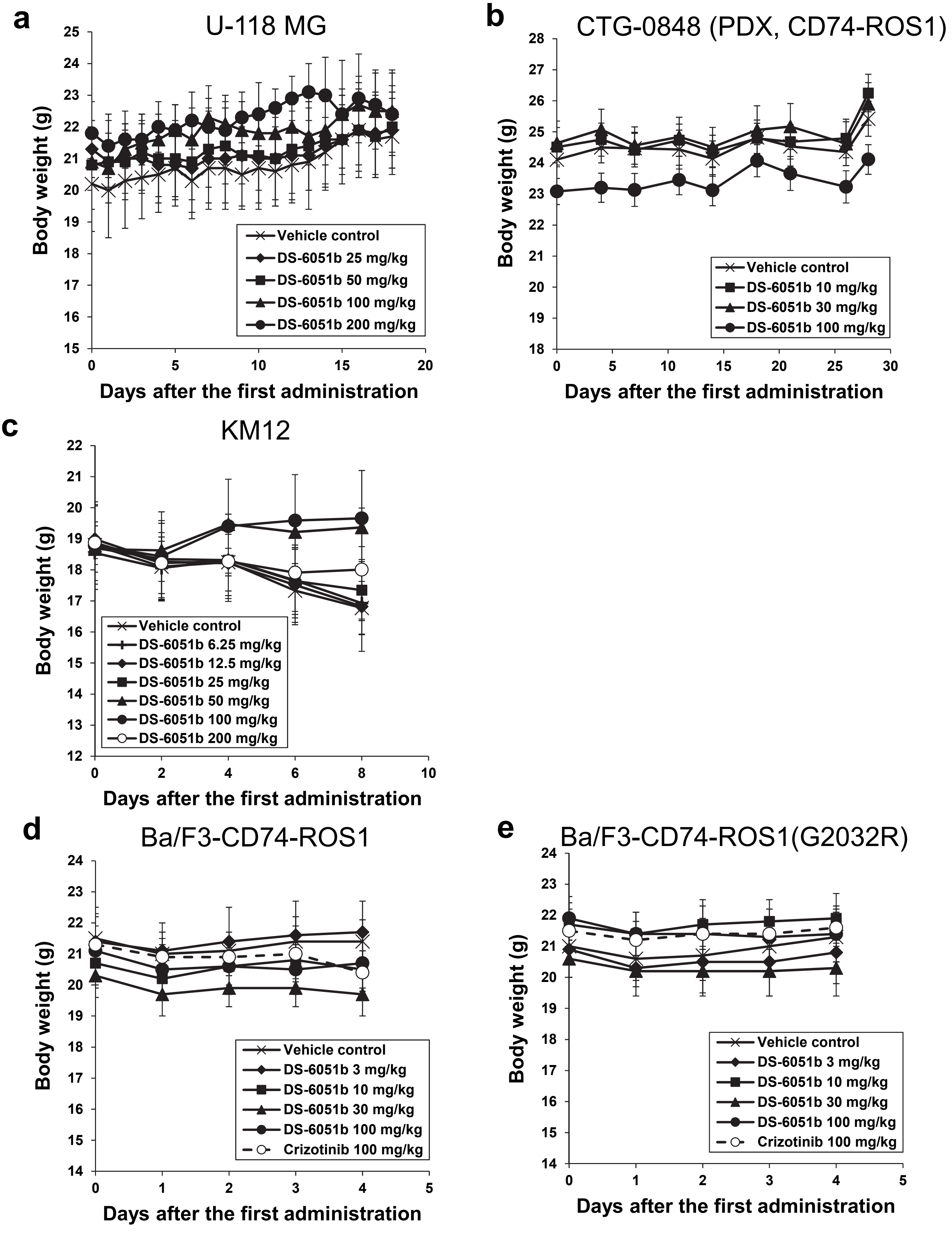

Supplementary Figure 5. DS-6051b did not induced body weight loss in mouse xenograft models.
(a-e) Mice bearing U118MG (a), CTG-0848 (PDX, CD74-ROS1 positive NSCLC)(b), KM12 (c), BaF3-CD74-ROS1-WT (d) or -G2032R (e) were orally administered DS-6051b at indicated doses, or crizotinib at 100 mg/kg, and the body weight changes were measured everyday, every other day, or twice a week.

Supplementary Figure 6

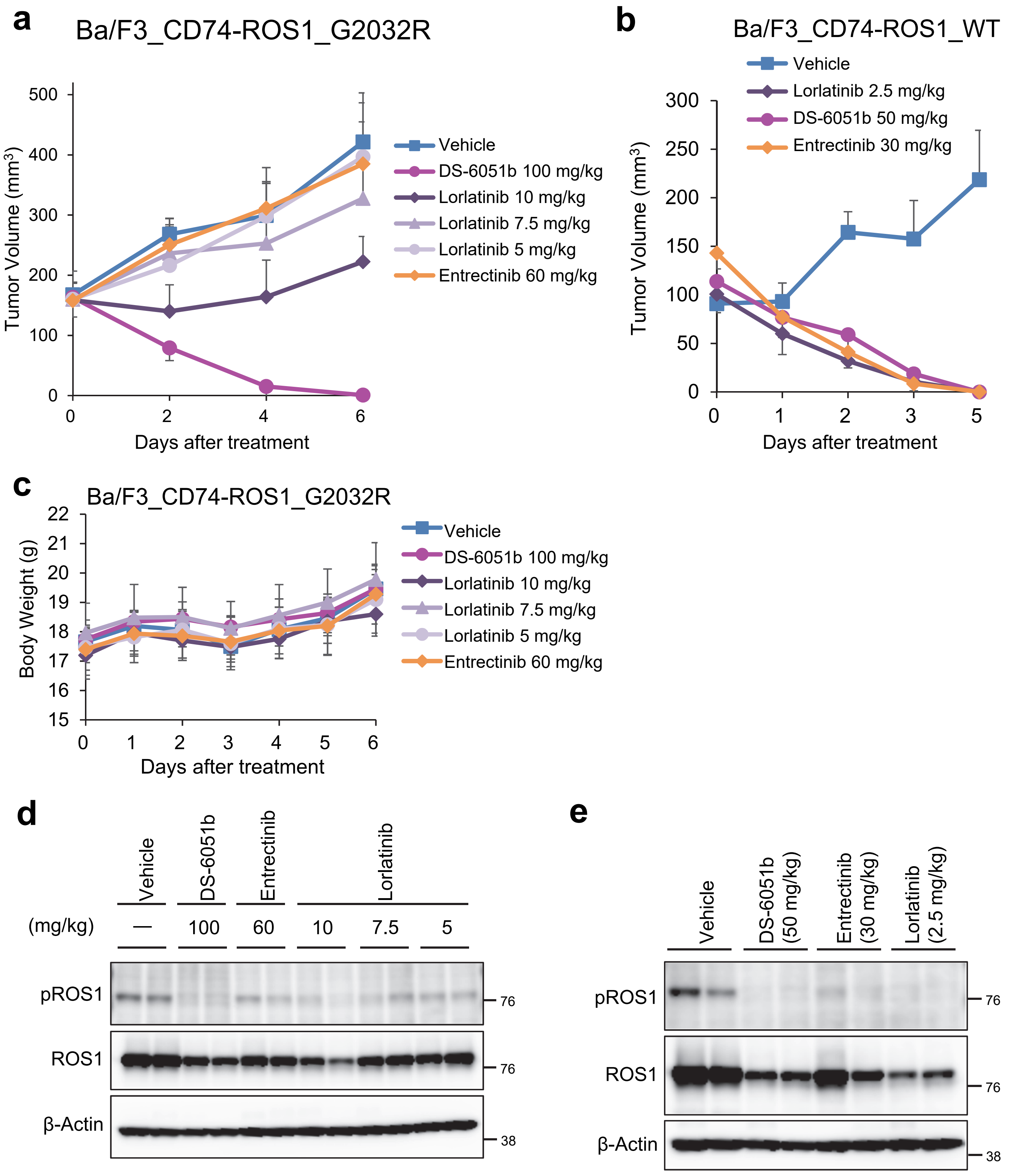

Supplementary Figure 6. DS-6051b but not lorlatinib or entrectinib induce tumor regression in Ba/F3-CD74-ROS1-G2032R mutant xenograft model

(a-c) BaF3-CD74-ROS1 -G2032R (a) or -WT (b) cells were subcutaneously implanted into Balb-c nu/nu mice. DS-6051b (50 or 100 mg/kg), lorlatinib (2.5, 5, 7.5, or 10 mg/kg), enrtectinib (30 or 60 mg/kg) or vehicle was treated once daily by oral gavage for 4 days. Results in (a,b) are indicated as mean ± SD of the tumor volume of each group (N = 6). And the body weight changes were measured everyday (c). (d, e) Mice bearing BaF3-CD74-ROS1-G2032R (d) or Ba/F3-CD74-ROS1-WT (e) were orally administered DS-6051b, entrectinib, and lorlatinib as indicated doses once a day. 3 hr after the second treatment, the tumors were collected and lysed. Phospho-ROS1 and ROS1 were detected by immunoblot analysis using corresponding antibodies.

Supplementary Figure 7

a

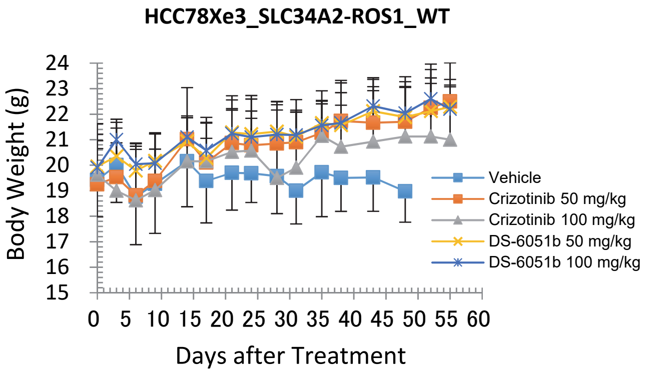

b

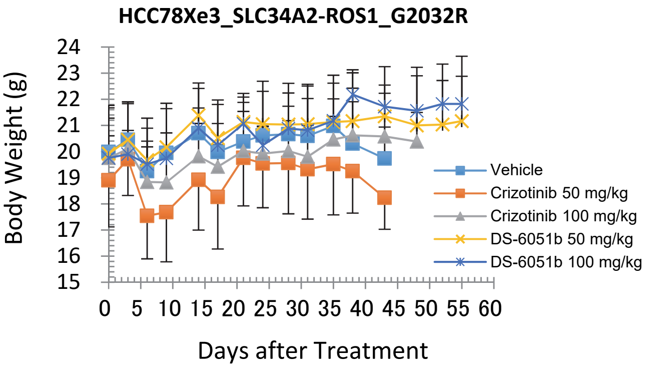

Supplementary Figure 7. DS-6051b did not induced body weight loss in mouse xenograft models.
(a, b) Mice bearing with SLC34A2-ROS1-WT (a) or -G2032R (b) were orally administered DS-6051b (50 or 100 mg/kg), or crizotinib (50 or 100 mg/kg), and the body weight changes were measured twice a week.

Supplementary Figure 8

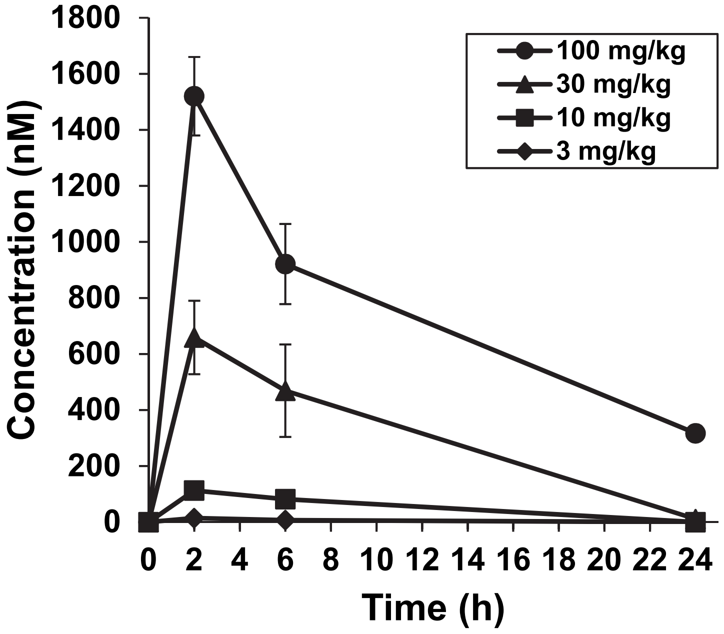

Supplementary Figure 8. PK profile of DS-6051b as a free base in nude mice
The indicated dose of DS-6051b as a free base was administered to nude mice. Blood samples were collected at 2, 6 and 24 h post-dosing and centrifuged to separate the supernatant as plasma. Plasma concentration of DS-6051b was quantified by LC-MS/MS system. Results are indicated as mean ± SD of the concentration of each group (N = 3).

Supplementary Figure 9

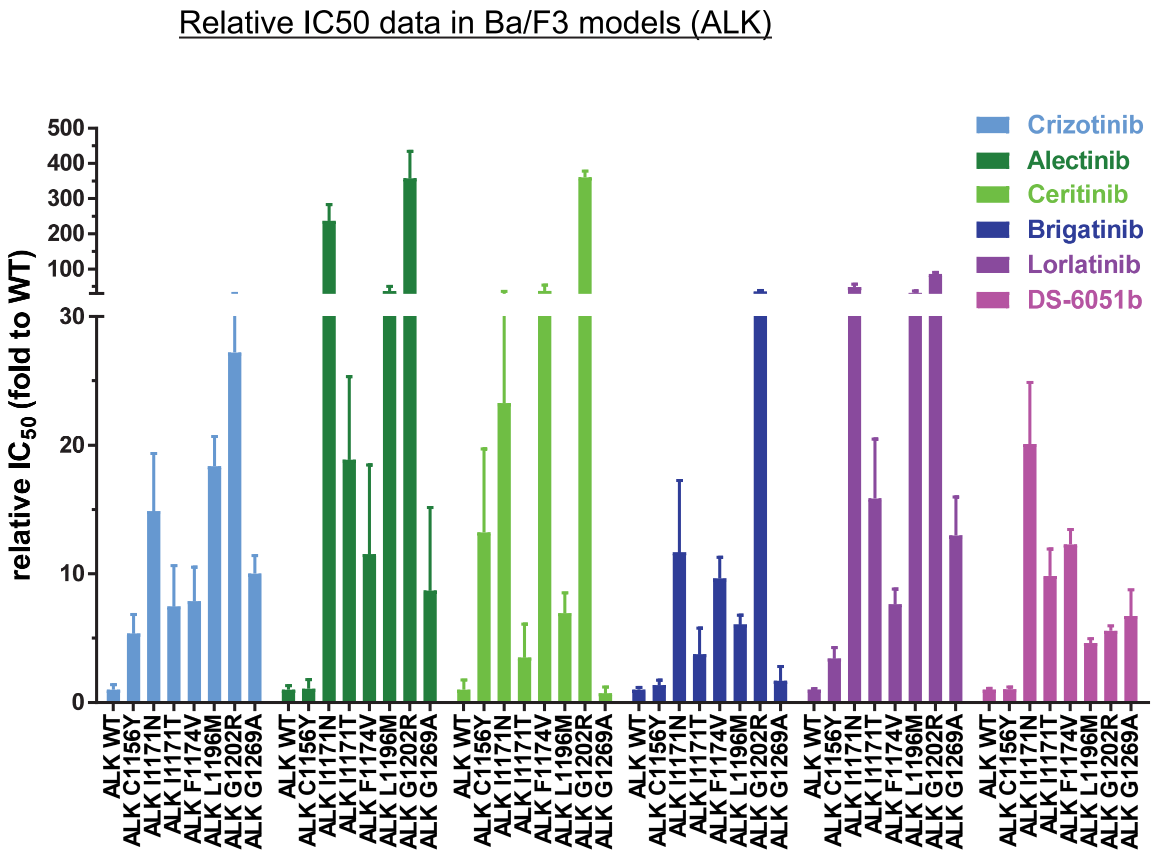

Supplementary Figure 9. DS-6051b was moderately active against WT but not resistant mutant ALK-rearranged cancers

Sensitivity of EML4-ALK (WT and mutants)–induced Ba/F3 cells to the indicated inhibitors including DS-6051b. The cells were treated with a range of inhibitor doses for 72 h. Cell viability was assessed using CellTiter-Glo assay. The calculated IC50 was shown in a bar graph.

Supplementary Figure 10

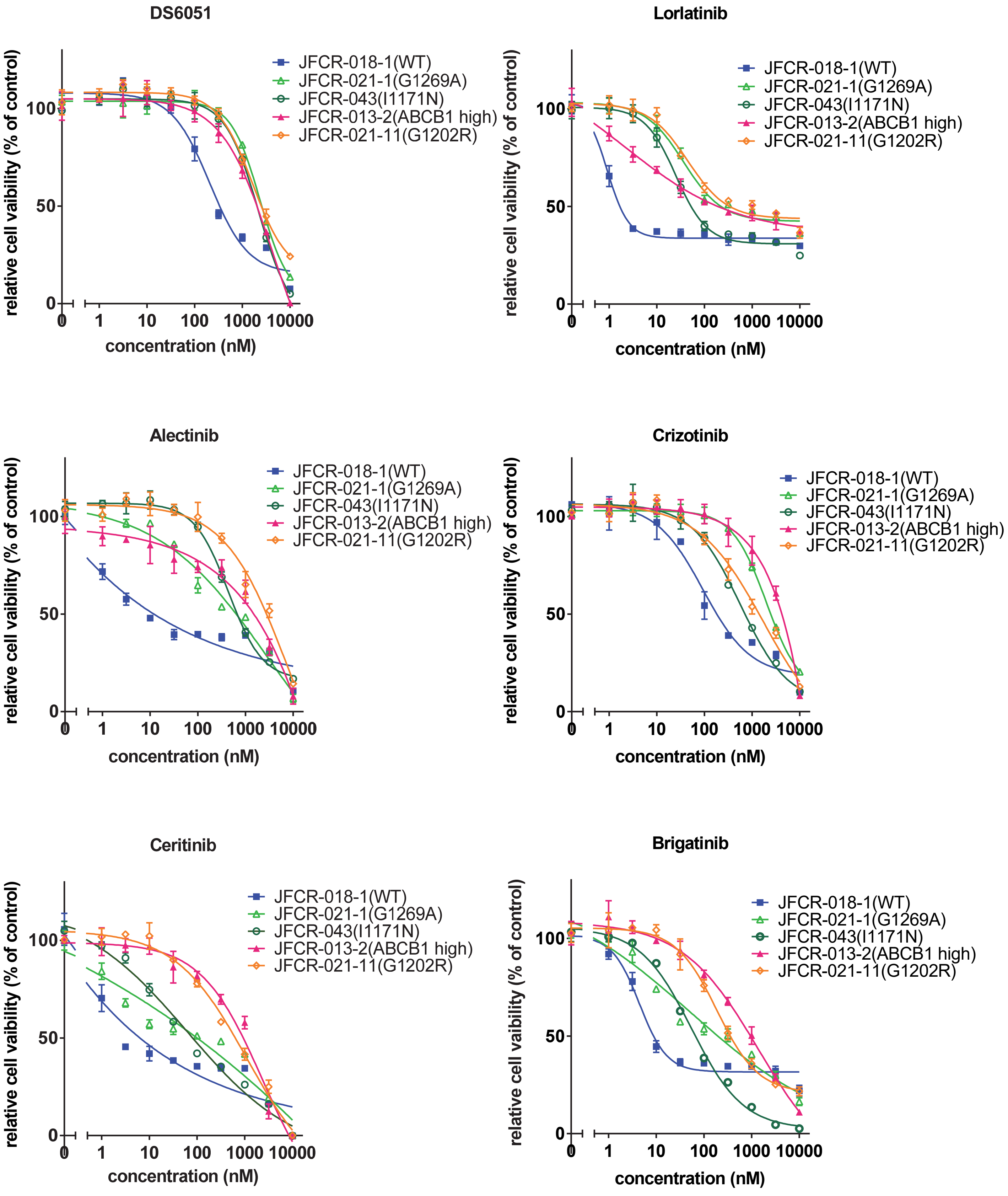

Supplementary Figure 10. DS-6051b was moderately active against ALK rearranged NSCLC cell line models

(a–f) Sensitivity of JFCR-018-1 (EML4-ALK-WT), JFCR-021-1 (G1269A), JFCR-043 (I1171N), JFCR-013-2 (ABCB1 overexpression), JFCR-021-11 (G1202R mutant) patient derived cells were treated with the indicated ROS1 or ALK inhibitors, such as DS-6051b, crizotinib, lorlatinib, alectinib, ceritinib, or brigatinib. The cells were treated with a range of inhibitor doses for 72 h. Cell viability was assessed using CellTiter-Glo assay.

Supplementary Figure 11

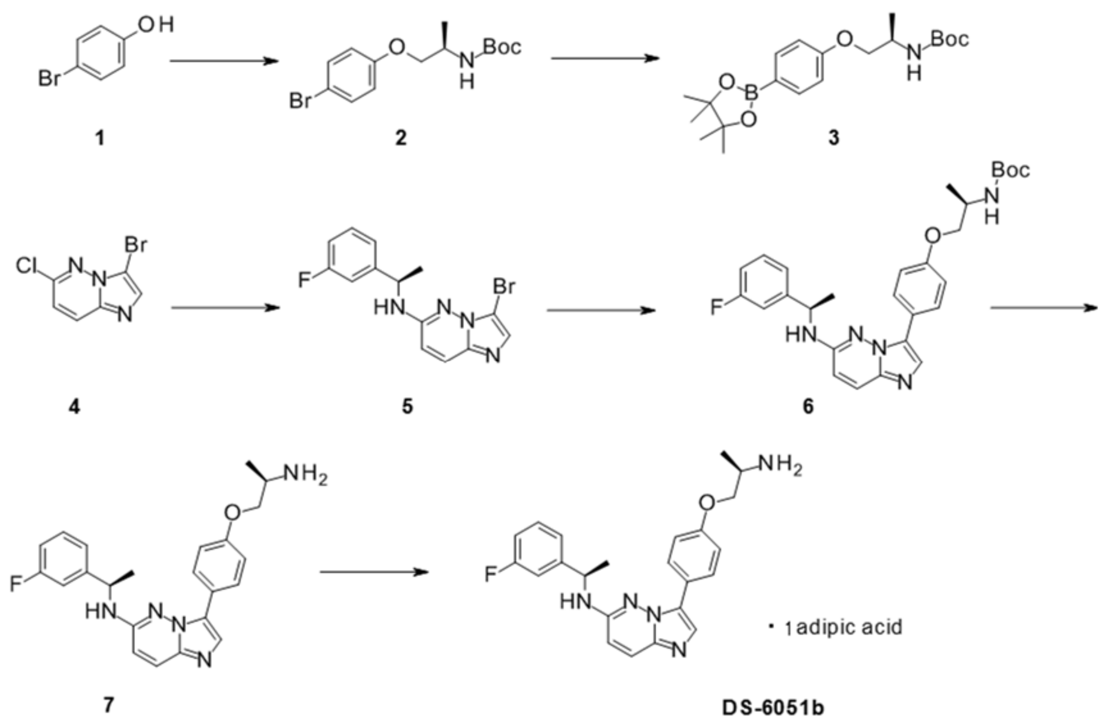

Supplementary Figure 11. Synthetic scheme of DS-6051b.

***tert*-butyl [(2*R*)-1-(4-bromophenoxy)propan-2-yl]carbamate (2)**

To a solution of 4-bromophenol **1** (2.60 g) and tert-butyl [(2R)-1-hydroxypropan-2-yl]carbamate (5.0 g) in tetrahydrofuran (50 ml), triphenylphosiphine (4.46 g) and a solution of diisopropyl azodicarboxylate (3.85 mL) in tetrahydrofuran (10 ml) were added, and the mixture was heated to reflux for 1 hour. After cooling, the reaction solution was concentrated under reduced pressure. The obtained residue was purified by silica gel column chromatography (n-hexane-ethyl acetate) to obtain the title compound (2.61 g).

^{1}H-NMR (400 MHz, $CDCl_3$) δ: 1.28 (3H, d, J = 6.7 Hz), 1.56 (3H, s), 3.89 (2H, d, J = 4.2 Hz), 3.98-4.09 (1H, m), 4.73 (1H, s), 6.76-6.81 (2H, m), 7.35-7.39 (2H, m).

***tert*-Butyl N-[(1*R*)-1-Methyl-2-[4-(4,4,5,5-tetramethyl-1,3,2-dioxaborolan-2-yl)phenoxy]ethyl]carbamate (3)**

To a solution of compound **2** (0.66 g) in 1,4-dioxane (10.0 ml), bis(pinacolato)diborane (0.559 g), potassium acetate (0.785 g), and [1,1'-bis(diphenylphosphino)ferrocene]dichloropalladium(II)-dichloromethane adduct (0.163 g) were added, and the mixture was stirred at 80°C for 1 hour under the nitrogen atmosphere. After cooling, ethyl acetate was added to the reaction solution, and insoluble matter was filtered off. The filtrate was concentrated under reduced pressure. Then, the obtained residue was purified by silica gel column chromatography (n-hexane-ethyl acetate) to obtain the title compound (0.394 g).

^{1}H-NMR (400 MHz, $CDCl_3$) δ: 1.29 (3H, d, J = 6.7 Hz), 1.33 (12H, s), 1.45 (9H, s), 3.94 (2H, d, J = 3.6 Hz), 4.06 (1H, br s), 4.78 (1H, br s), 6.89 (2H, d, J = 8.5 Hz), 7.74 (2H, d, J = 8.5 Hz).

3-Bromo-N-[(1*R*)-1-(3-fluorophenyl)ethyl]imidazo[1,2-*b*]pyridazin-6-amine (5)

3-Bromo-6-chloroimidazo[1,2-b]pyridazine **4** (16.0 g), (1*R*)-1-(3-fluorophenyl)ethan-1-amine (12.4 g), and potassium fluoride (8.13 g) were dissolved in dimethyl sulfoxide (200 ml), and the solution was stirred at 130°C for 24 hours. After cooling, water was added to the reaction solution, followed by extraction with ethyl acetate. The extract was washed with saturated saline and then dried over anhydrous magnesium sulfate. The solvent was distilled off under reduced pressure. The obtained residue was purified by silica gel column chromatography (hexane-ethyl acetate) to obtain the title compound (15.8 g).

^{1}H-NMR (400 MHz, $CDCl_3$) δ: 1.61 (3H, d, J = 6.9 Hz), 4.83 (1H, d, J = 6.3 Hz), 5.01-5.06 (1H, m), 6.42 (1H, d, J = 9.7 Hz), 6.93-6.97 (1H, m), 7.17 (1H, dt, J = 9.9, 2.1 Hz), 7.24 (1H, d, J = 7.4 Hz), 7.30 (1H, td, J = 7.9, 5.9 Hz), 7.46 (1H, s), 7.57 (1H, t, J = 4.6 Hz).

***tert*-Butyl N-[(1*R*)-2-[4-[6-[[(1*R*)-1-(3-Fluorophenyl)ethyl]amino]imidazo[1,2-*b*]pyridazin-3-yl]phenoxy]-1-methylethyl]carbamate (6)**

1,4-Dioxane (25 ml) and water (5 ml) were added to compound **5** (0.34 g), compound **3** (0.39 g), potassium carbonate (0.55 g), and [1,1'-bis(diphenylphosphino)ferrocene]dichloropalladium(II)-dichloromethane adduct (81 mg), and the mixture was heated to reflux for 1.5 hours under the nitrogen atmosphere. After cooling, water was added to the reaction solution, followed by extraction with ethyl acetate. The extract was washed with saturated saline and then dried over anhydrous magnesium sulfate. The solvent was distilled off under reduced pressure. The obtained residue was purified by silica gel column chromatography (ethyl acetate-methanol) to obtain the title compound (0.39 g).

^{1}H-NMR (400 MHz, $CDCl_3$) δ: 1.33 (3H, d, J = 6.7 Hz), 1.48 (9H, s), 1.57 (3H, d, J = 6.7 Hz), 3.99 (2H, d, J = 3.6 Hz), 4.10-4.13 (1H, m), 4.68 (1H, d, J = 4.8 Hz), 4.83 (1H, br s), 4.92 (1H, dq, J = 4.8, 6.7 Hz), 6.47 (1H, d, J = 9.7 Hz), 6.90 (2H, d, J = 9.1 Hz), 6.97 (1H, td, J = 8.5, 2.4 Hz), 7.11-7.14 (1H, m), 7.20 (1H, d, J = 7.9 Hz), 7.36 (1H, td, J = 7.9, 6.0 Hz), 7.61-7.62 (2H, m), 7.67 (2H, d, J = 9.7 Hz).

3-[4-[(2*R*)-2-Aminopropoxy]phenyl]-N-[(1*R*)-1-(3-fluorophenyl)ethyl]imidazo[1,2-*b*]pyridazin-6-amine (7)

To a solution of compound **6** (0.39 g) in methanol (3 ml), a solution of 4 N hydrochloric acid in 1,4-dioxane (8 ml) was added, and the mixture was stirred at room temperature for 40 minutes. The reaction solution was concentrated under reduced pressure. A 1 N aqueous sodium hydroxide solution was added to the obtained residue, followed by extraction with chloroform-methanol. The extract was

washed with saturated saline and then dried over anhydrous sodium sulfate. The solvent was distilled off under reduced pressure. The obtained residue was purified by silica gel column chromatography (basic silica gel, chloroform-methanol) to obtain the title compound (0.09 g).

^{1}H-NMR (400 MHz, DMSO-d_6) δ: 1.08 (3H, d, J = 6.0 Hz), 1.48 (3H, d, J = 7.3 Hz), 1.65 (2H, br s), 3.12-3.21 (1H, m), 4.19-4.19 (2H, m), 4.80-4.89 (1H, m), 6.77 (1H, d, J = 9.7 Hz), 6.93 (2H, d, J = 9.1 Hz), 7.01-7.06 (1H, m), 7.22-7.29 (2H, m), 7.37-7.44 (1H, m), 7.62 (1H, d, J = 6.0 Hz), 7.70-7.77 (4H, m); ESI-MS (m/z) : 406 $(M+H)^+$.

3-[4-[(2*R*)-2-Aminopropoxy]phenyl]-N-[(1*R*)-1-(3-fluorophenyl)ethyl]imidazo[1,2-*b*]pyridazin-6-amine Monoadipate (DS-6051b)

To compound **7** (500 mg), adipic acid (181 mg) and 1-propanol (5 ml) were added at room temperature. The mixture was stirred at 40°C for 24 hours and further stirred at room temperature for 30 minutes. The deposited solid was collected by filtration to obtain the title compound (622 mg).

^{1}H-NMR (400 MHz, DMSO-d_6) δ: 1.11 (3H, d, J = 6.0 Hz), 1.46-1.51 (4H, m), 1.48 (3H, d, J = 7.3 Hz), 2.15-2.21 (4H, m), 3.18-3.27 (1H, m), 3.77-3.86 (2H, m), 4.80-4.88 (1H, m), 6.77 (1H, d, J = 9.7 Hz), 6.93 (2H, d, J = 9.1 Hz), 7.01-7.06 (1H, m), 7.22-7.29 (2H, m), 7.36-7.43 (1H, m), 7.61 (1H, d, J = 6.0 Hz), 7.70-7.77 (4H, m); ^{13}C-NMR (125 MHz, DMSO-d_6) δ: 18.59, 23.48, 24.37, 34.09, 45.71, 50.66, 72.79, 111.38, 112.44, 113.14, 114.31, 121.60, 121.79, 125.63, 126.54, 127.03, 128.79, 130.13, 136.54, 148.77, 152.10, 157.40, 162.29, 174.69; ESI-MS (m/z) : 406 $(M+H)^+$; Anal. Calcd for $C_{23}H_{24}FN_5O \cdot C_6H_{10}O_4$: C, 63.14; H, 6.21; N, 12.70. Found: C, 62.97; H, 6.29; N, 12.59; $[\alpha]_D^{23}$ = +43.3° (c=0.80, MeOH).

LC-MS and NMR spectra of DS-6051b were shown in the figures below.

Instrument : Agilent
Method : MS100-1000.M
Date : 16 May 19 10:23 am +0900

LC-MS DS-6051b

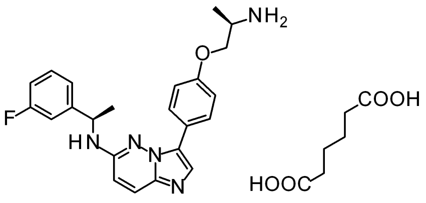

[UV]
UV File : DAD1A.CH
Max Intensity : 6516916 , Min Intensity : -762589 , Scan# : 975 , Ret. time : 1.87 min.

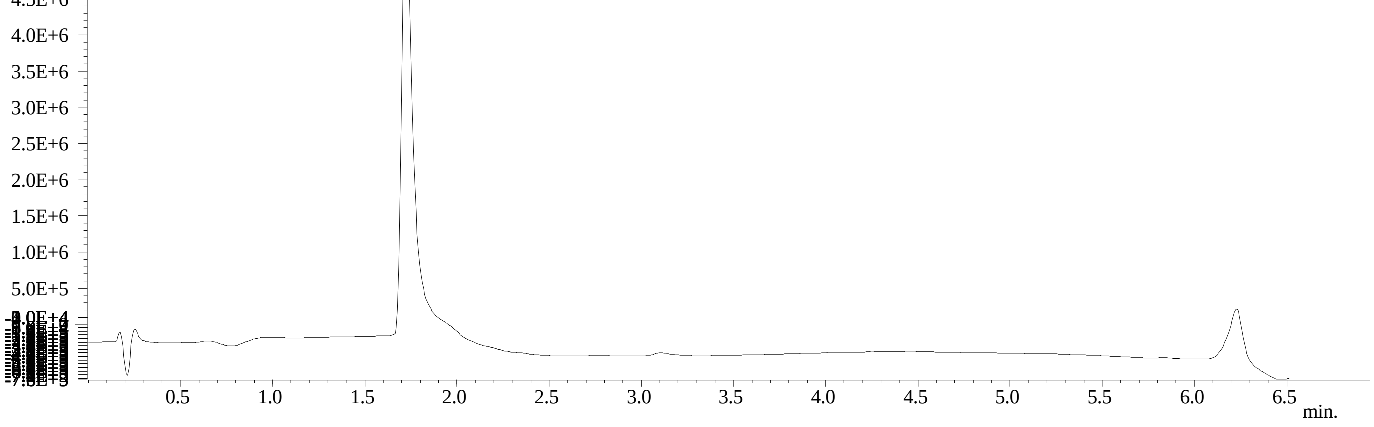

[Total Ion Chromatogram]
Max Intensity : 52261 at scan# : 138 / Ret. time : 1.87 min.
Total scans : 489

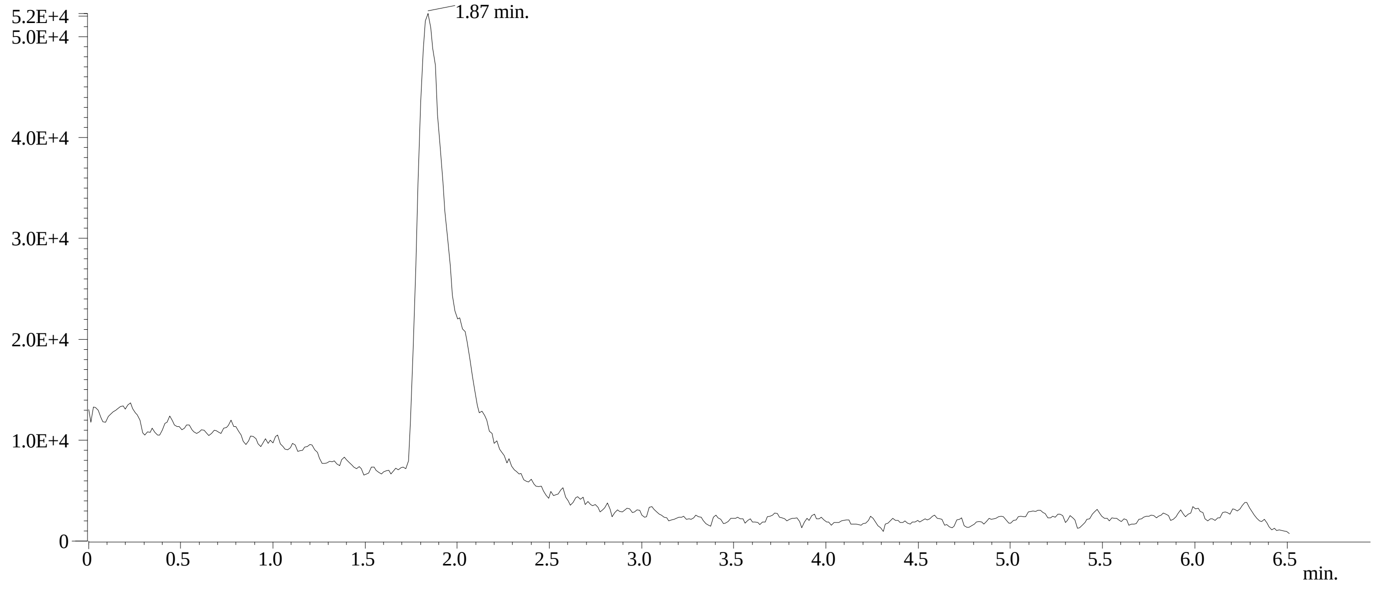

[Mass Spectrum]
Scan# : 138 Ret. time : 1.87 min.
Max Intensity : 18376

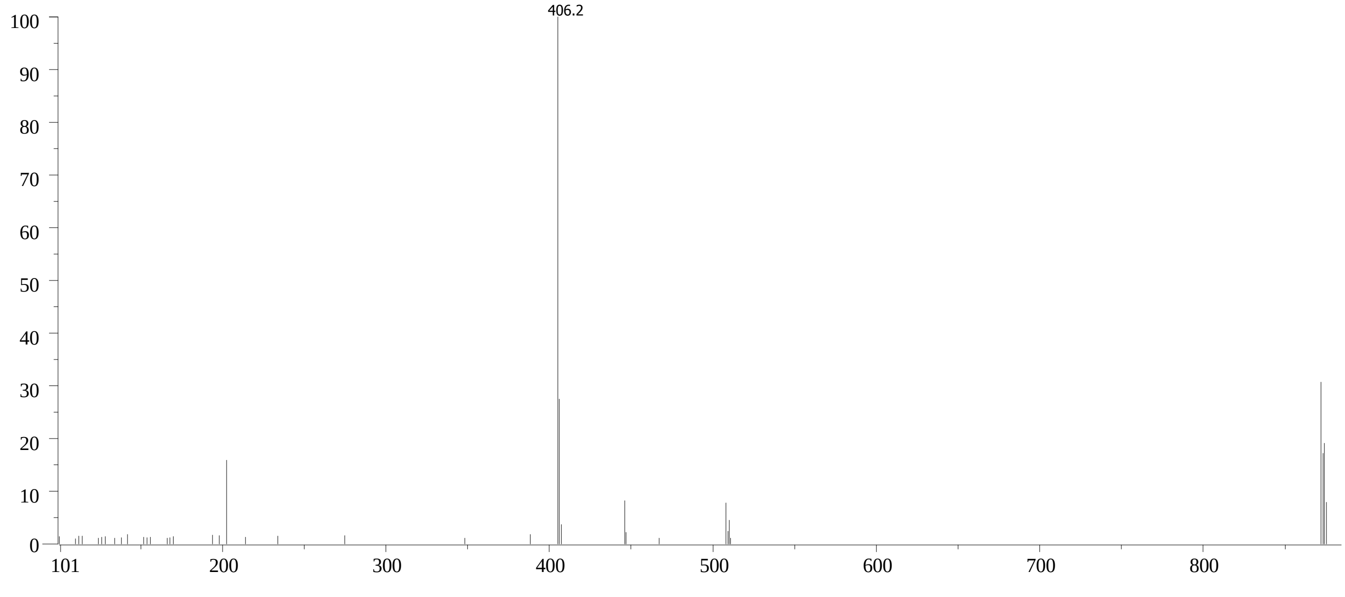

^{1}H-NMR DS-6051b

```
DATIM  2012-09-03 14:01:12
OBNUC  1H
EXMOD  proton.jxp
OBFRQ        395.88 MHz
OBSET          6.28 KHz
OBFIN          0.87 Hz
POINT         16384
FREQU       9904.91 Hz
SCANS            32
ACQTM        1.6541 sec
PD           2.0000 sec
PW1            4.96 usec
IRNUC  1H
CTEMP          24.1 c
SLVNT  DMSO
EXREF          0.00 ppm
BF             0.12 Hz
RGAIN            50
```

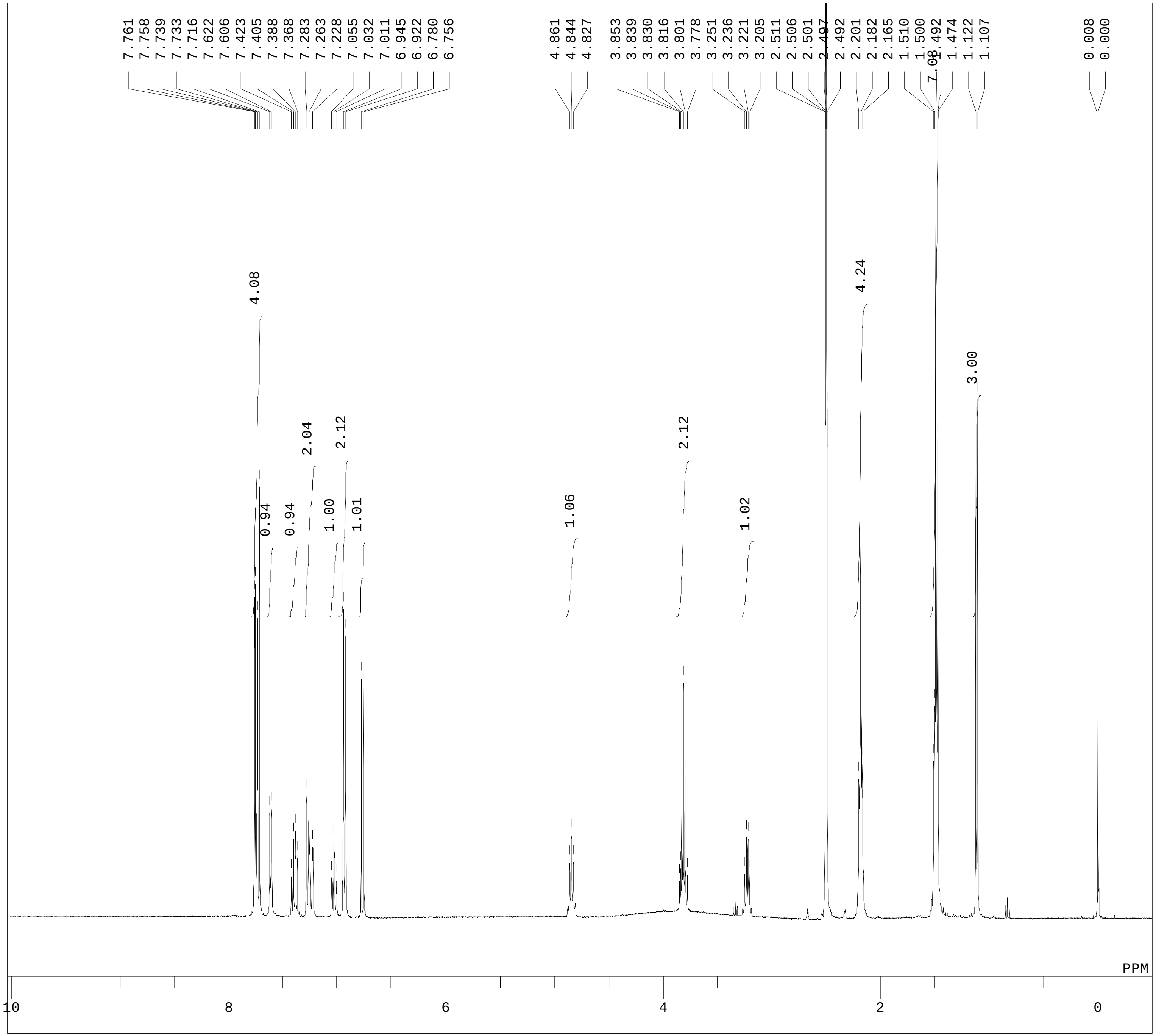

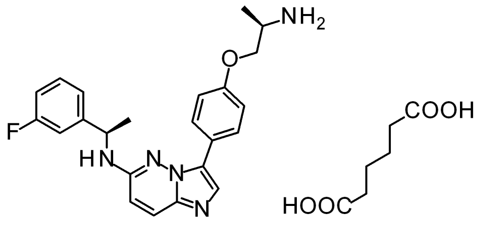

^{13}C-NMR DS-6051b ·(20mg/0.5mL,DMSO-d6;13C complete 1H and 19F CPD;2019/05/09)

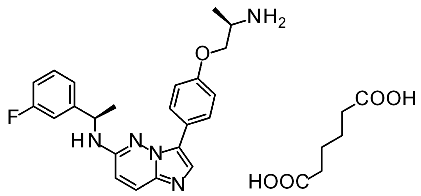

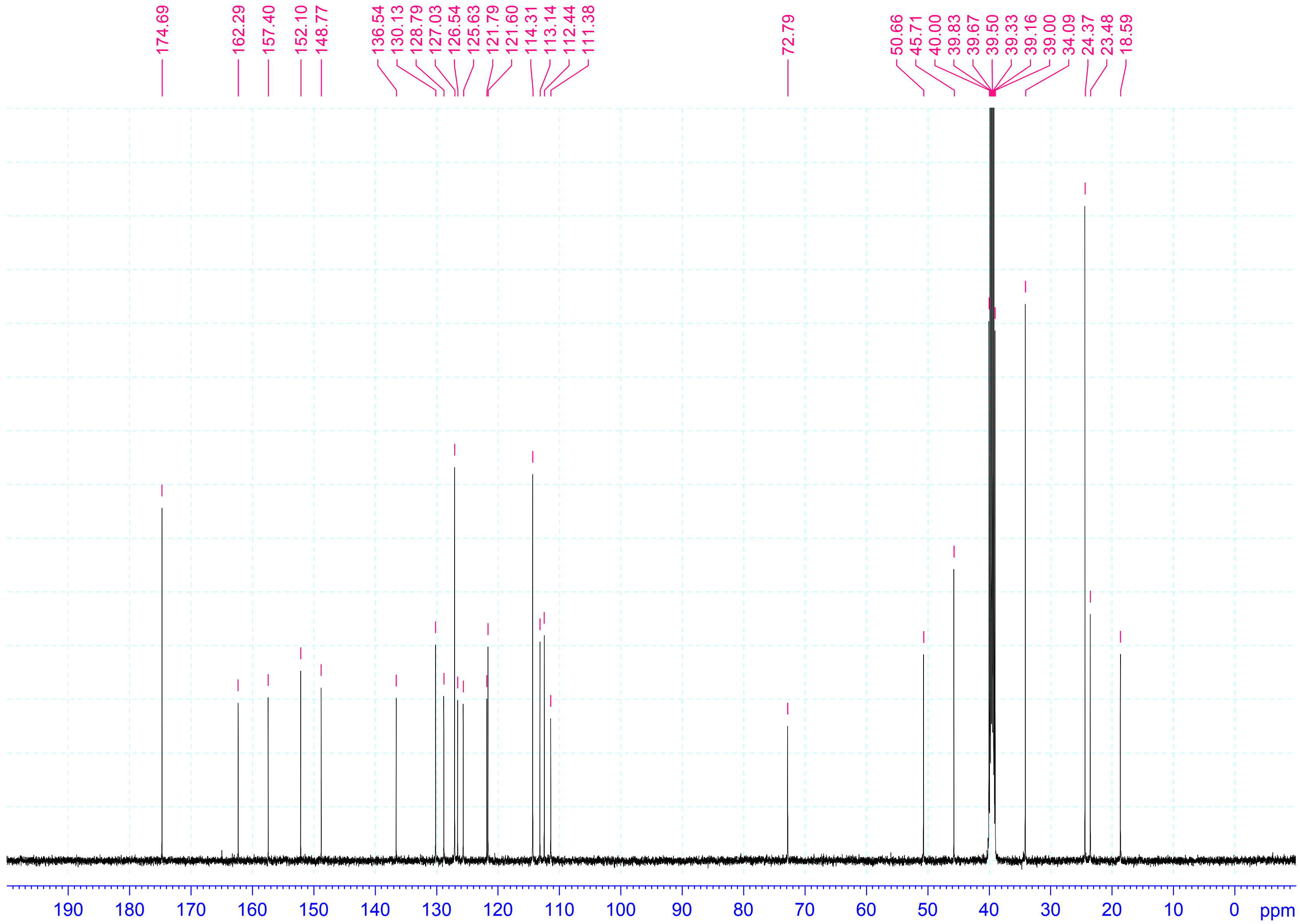

Current Data Parameters
NAME NA1010420_TXO
EXPNO 1
PROCNO 1

F2 - Acquisition Parameters
Date_ 20190509
Time 23.36
INSTRUM spect
PROBHD 5 mm PATXO 19F
PULPROG zg30f2cpdf3cpd.m
TD 65536
SOLVENT DMSO
NS 4096
DS 64
SWH 31250.000 Hz
FIDRES 0.476837 Hz
AQ 1.0485760 sec
RG 193.07
DW 16.000 usec
DE 8.00 usec
TE 304.8 K
D1 3.00000000 sec
D11 0.03000000 sec
D12 0.00002000 sec
TD0 1

======== CHANNEL f1 ========
SFO1 125.7716224 MHz
NUC1 13C
P1 10.00 usec
PLW1 80.00000000 W

======== CHANNEL f2 ========
SFO2 500.1320005 MHz
NUC2 1H
CPDPRG[2 waltz16
PCPD2 80.00 usec
PLW2 20.00000000 W
PLW12 0.34452999 W
PLW13 0.22050001 W

======== CHANNEL f3 ========
SFO3 470.5412053 MHz
NUC3 19F
CPDPRG[3 bi_p5m4sp_4sp.2
P63 1500.00 usec
PLW3 30.00000000 W
PLW16 0.88099998 W
SPNAM[14 Crp80.1500.20
SPOAL14 0.500
SPOFFS14 0 Hz
SPW14 1.41999996 W
SPNAM[31 Crp80.1500.20
SPOAL31 0.500
SPOFFS31 0 Hz
SPW31 0.70999998 W

F2 - Processing parameters
SI 65536
SF 125.7578566 MHz
WDW EM
SSB 0
LB 1.00 Hz
GB 0
PC 1.40

Supplementary Table 1

a

Kinase	IC_{50} (nM)
ROS1	0.898
NTRK1	3.01
NTRK2	9.52
NTRK3	9.28
ACK	15.4
ALK	32.5
DDR1	24.7
DDR2	299
KIT	349
LTK	25.4
TXK	183

b

Kinase	% of inhibition
NTRK3	103.4
ALK	103.4
NTRK1	101.1
NTRK2	100.9
ROS	99.8
ACK	98.8
NuaK1	98.6
LTK	98.5
DDR1	98.2
JAK2	97.6
MUSK	96.7
DDR2	93.7
FAK	91.6
FMS	91.3

c

Cell Line	IC_{50} (nM)
Ba/F3-ETV6-NTRK1	18.7
Ba/F3-ETV6-NTRK2	20.6
Ba/F3-ETV6-NTRK3	8.4

Supplementary Table 1. Efficacy of the novel ROS1/NTRK inhibitor DS-6051b in vitro.
(a) In vitro kinase assay in the presence of DS-6051b and the indicated recombinant ROS1 or NTRK family kinases. The calculated IC50 values were indicated. (b) In vitro kinase assay in the presence of 40 nM of DS-6051b, the indicated recombinant family kinases, and ATP at the concentration of Km for ATP. % of inhibition was indicated. (c) Sensitivity of ETV6-NTRK1, 2, and 3 introduced Ba/F3 cells to DS-6051b. .

Supplementary Table 2

IC_{50} (nM)	Crizotinib	Alectinib	Ceritinib	Brigatinib	Lorlatinib	DS-6051b
ALK WT	12.33	1.38	0.44	1.89	1.00	46.64
ALK I1171N	183.33	327.27	10.23	22.06	48.35	938.10
ALK G1202R	335.60	493.43	158.53	68.75	85.05	260.67
ALK I1171T	92.14	26.08	1.54	7.10	15.81	459.33
ALK C1156Y	66.18	1.48	5.82	2.57	3.42	48.34
ALK F1174V	97.35	15.95	16.48	18.22	7.62	573.13
ALK L1196M	226.47	50.61	3.06	11.50	32.70	215.63
ALK G1269A	123.63	12.03	0.32	3.21	12.93	314.30

IC_{50} (nM)	Crizotinib	Alectinib	Ceritinib	AP26113	Lorlatinib	DS-6051b
LMNA-NTRK1	18.26	354.17	584.33	416.47	149.70	3.35
TPM3-NTRK1	21.43	284.27	596.23	926.23	168.07	2.97

Supplementary Table 2. IC50 values of DS-6051b and various ALK/ROS1 inhibitors to ALK or NTRK1 fusion gene induced Ba/F3 cells
Sensitivity of EML4-ALK (WT or resistant mutants), or NTRK1 fusion genes induced Ba/F3 cells to DS-6051b and the indicated inhibitors. The cells were treated with a range of inhibitor doses for 72 h. Cell viability was assessed using CellTiter-Glo assay. The calculated IC50 values were indicated.
