# Supplementary material for: The new-generation selective ROS1/NTRK inhibitor DS-6051b overcomes crizotinib resistant ROS1-G2032R mutation in preclinical models
Source: Nat Commun. 2019 Aug 9;10:3604. doi: 10.1038/s41467-019-11496-z (PMC6688997; doi:10.1038/s41467-019-11496-z)
Supplement: Supplementary file 2 — Description of Additional Supplementary Files [file 41467_2019_11496_MOESM2_ESM.pdf]

Description of Additional Supplementary Files

File name: Supplementary Data 1

Description: Revised Supplementary Data_in vitro kinase assay at KmATP: In vitro kinase assay data of DS-6051b against about 250 kinases at KmATP.

File name: Supplementary Data 2

Description: Revised supplementary Data_NGS data of PDX or PDC models: mutation and gene alterations (fusion genes) of PDX model and PDCs (Patient derived cell lines).
