# Supplementary material for: The new-generation selective ROS1/NTRK inhibitor DS-6051b overcomes crizotinib resistant ROS1-G2032R mutation in preclinical models
Source: Nat Commun. 2019 Aug 9;10:3604. doi: 10.1038/s41467-019-11496-z (PMC6688997; doi:10.1038/s41467-019-11496-z)

Supplementary Data: Original uncropped image of immunoblots (Fig 1f)

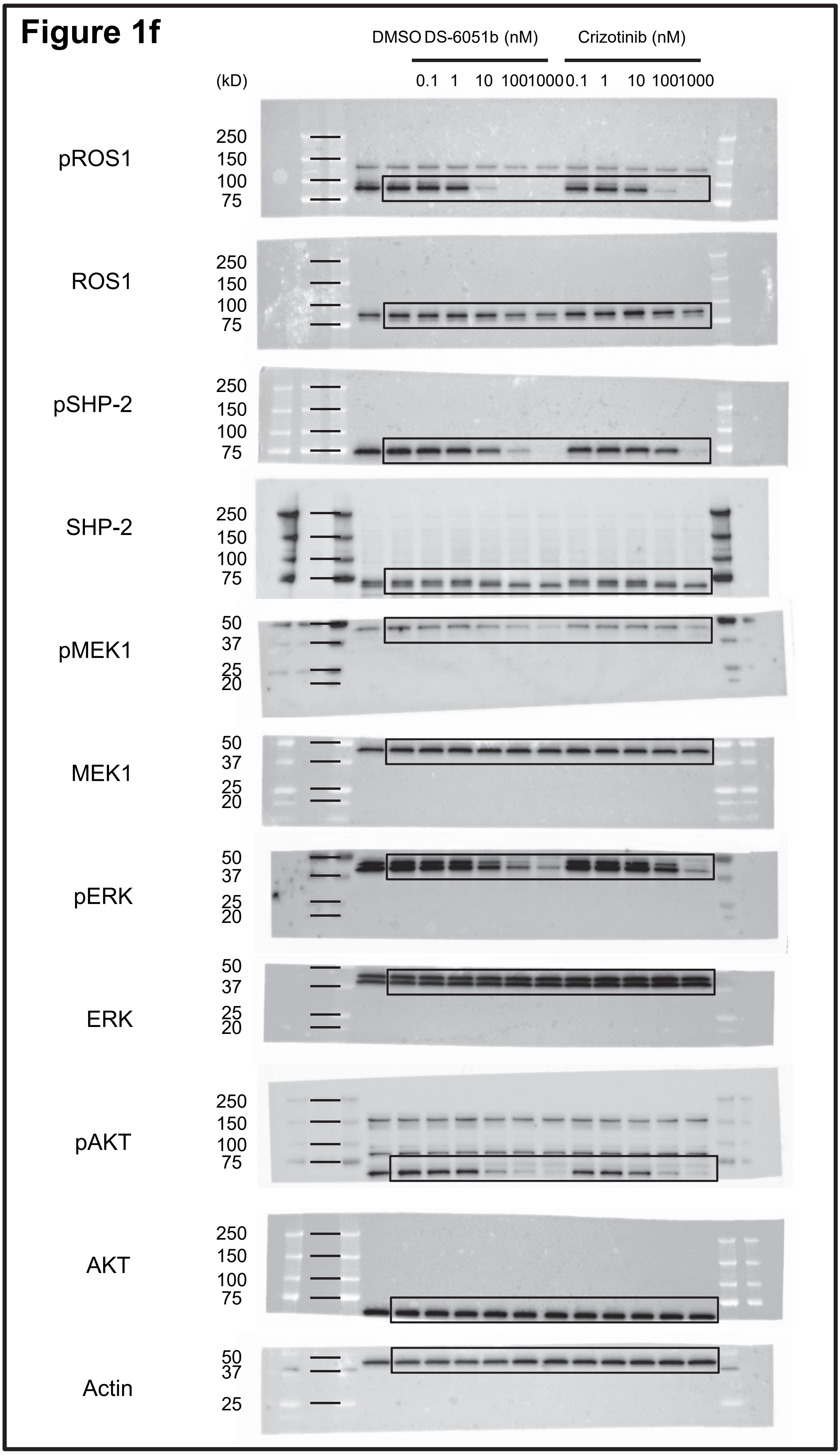

Supplementary Data: Original uncropped image of immunoblots (Fig 1g)

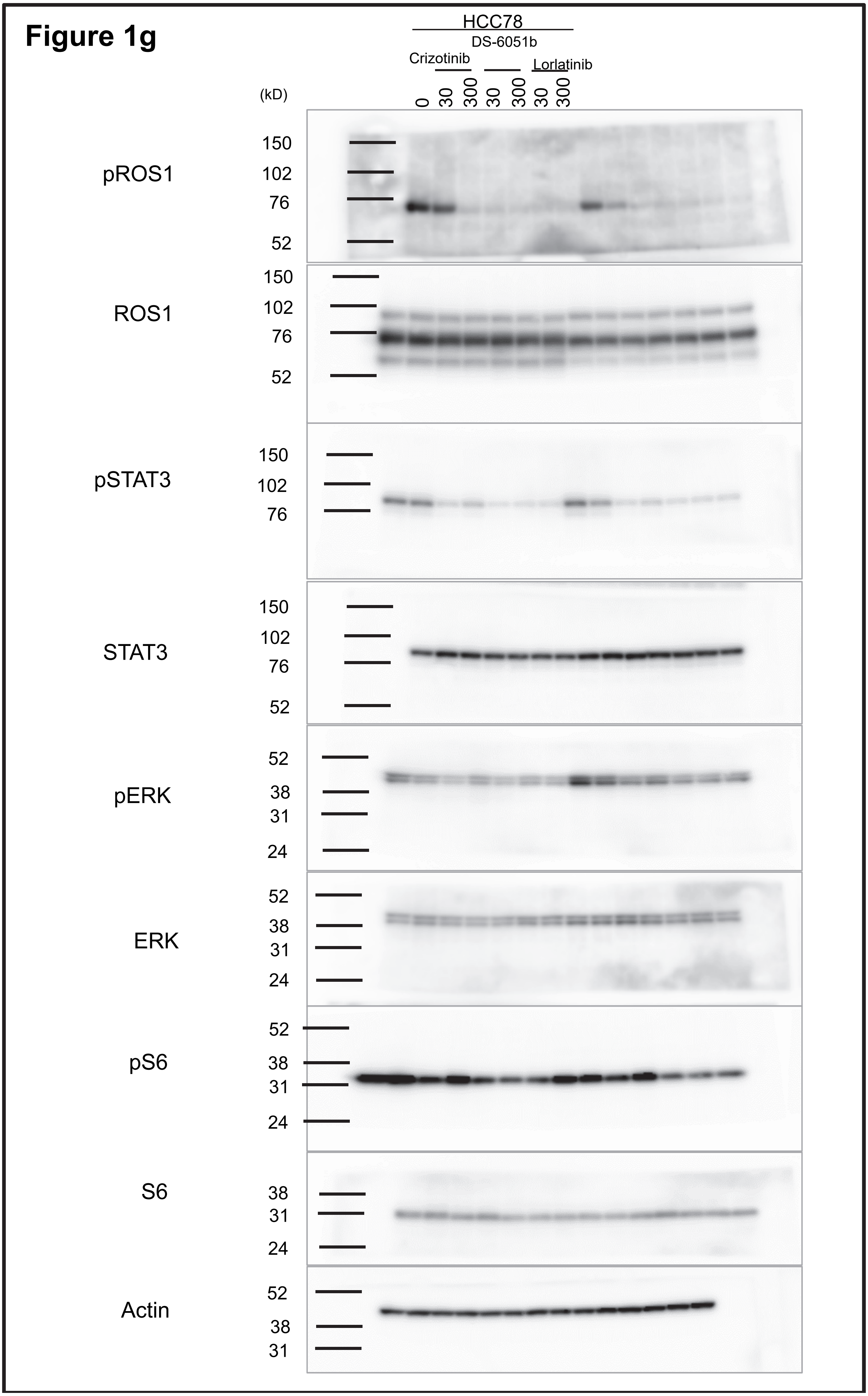

Supplementary Data: Original uncropped image of immunoblots (Fig 2d)

WB of JFCR-165

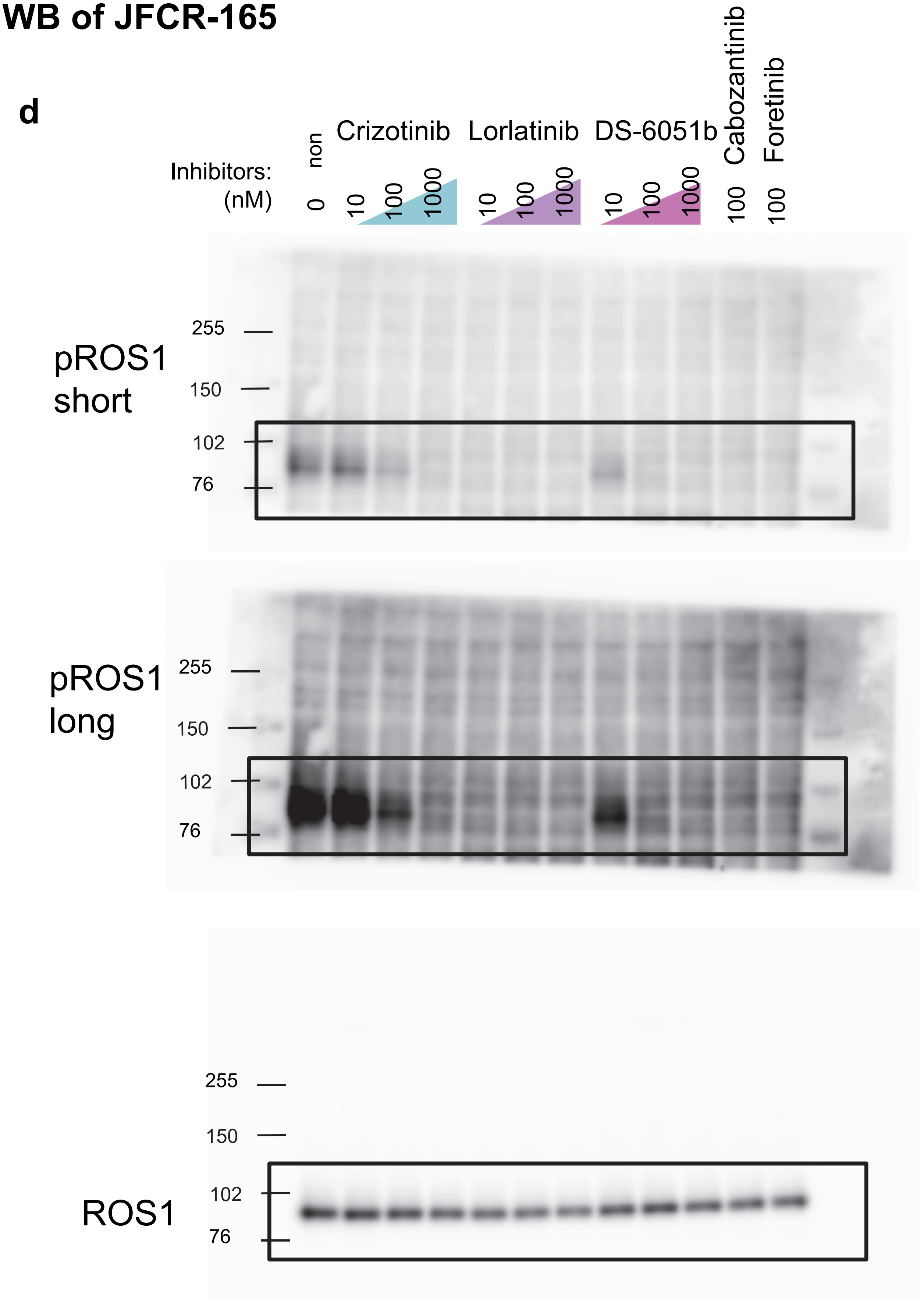

Supplementary Data: Original uncropped image of immunoblots (Fig 2d)

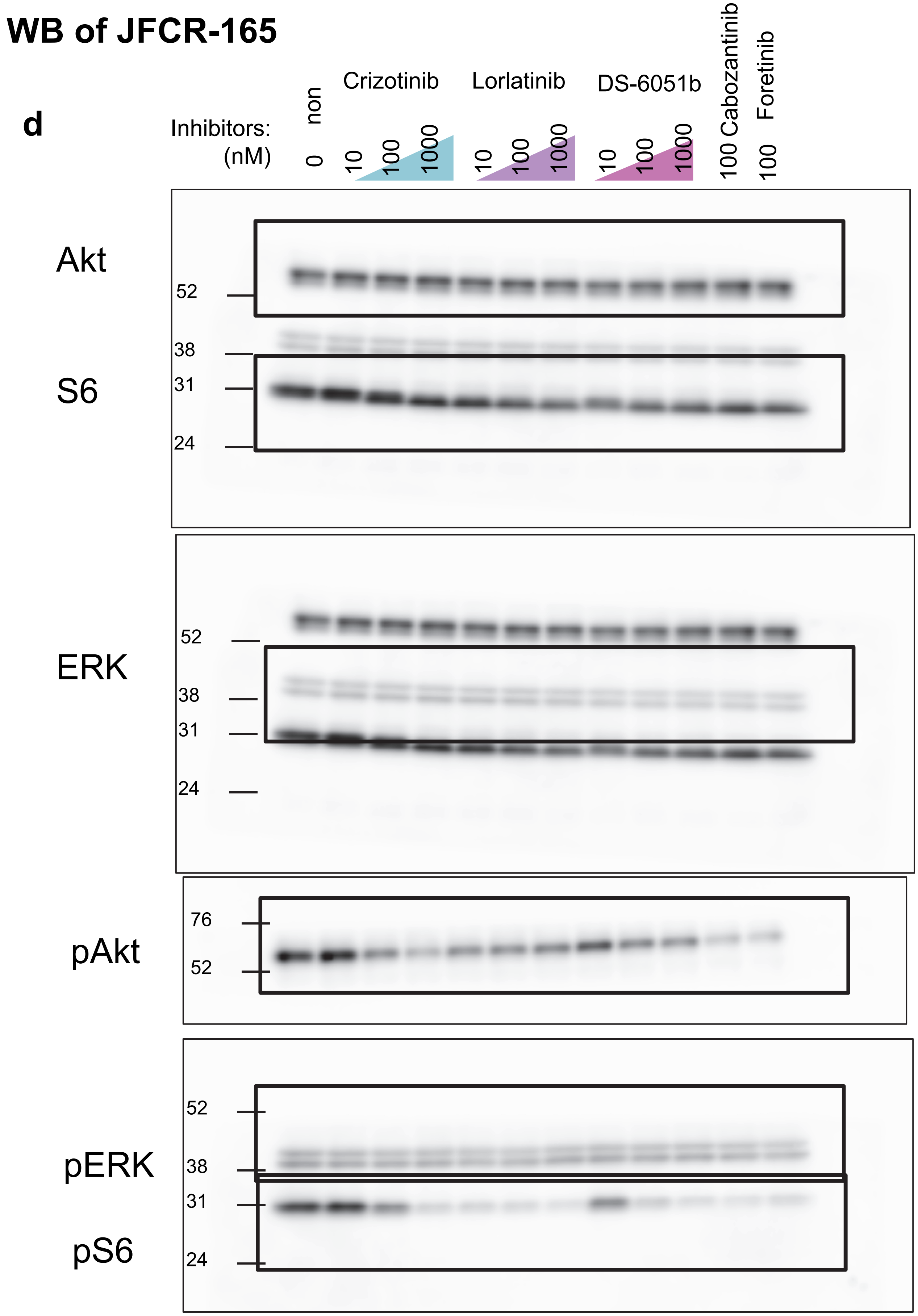

Supplementary Data: Original uncropped image of immunoblots (Fig 2d)

WB of JFCR-165

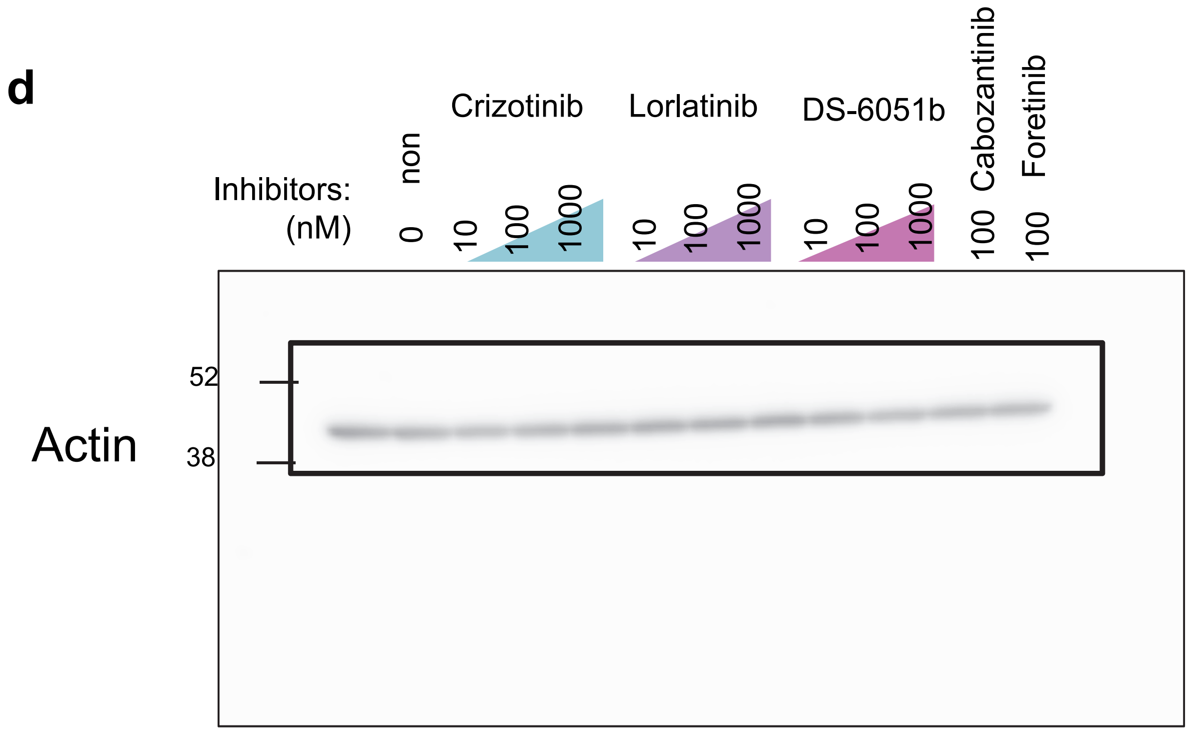

Supplementary Data: Original uncropped image of immunoblots (Fig 2e)

WB of JFCR-168

e

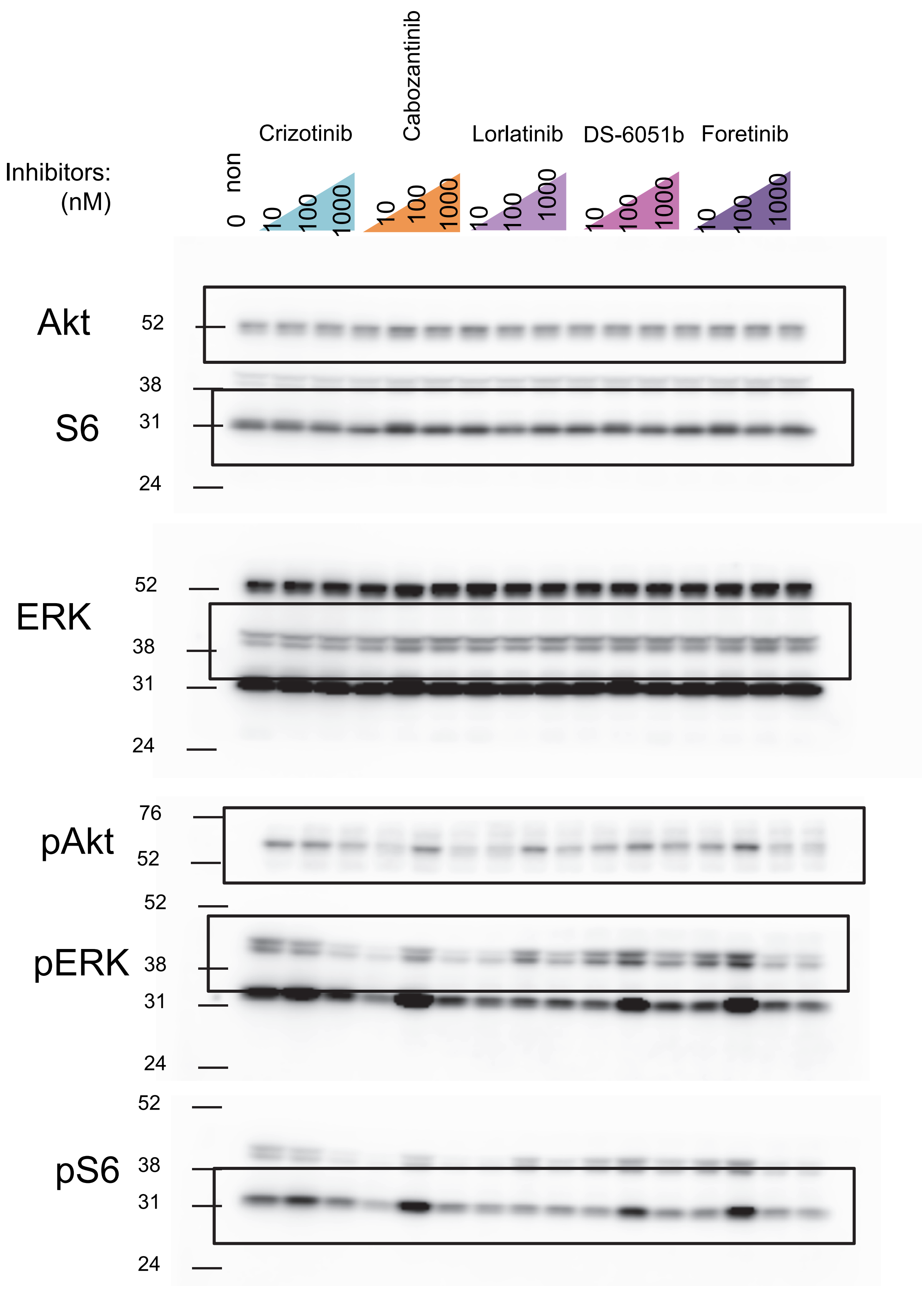

Supplementary Data: Original uncropped image of immunoblots (Fig 2e)

WB of JFCR-168

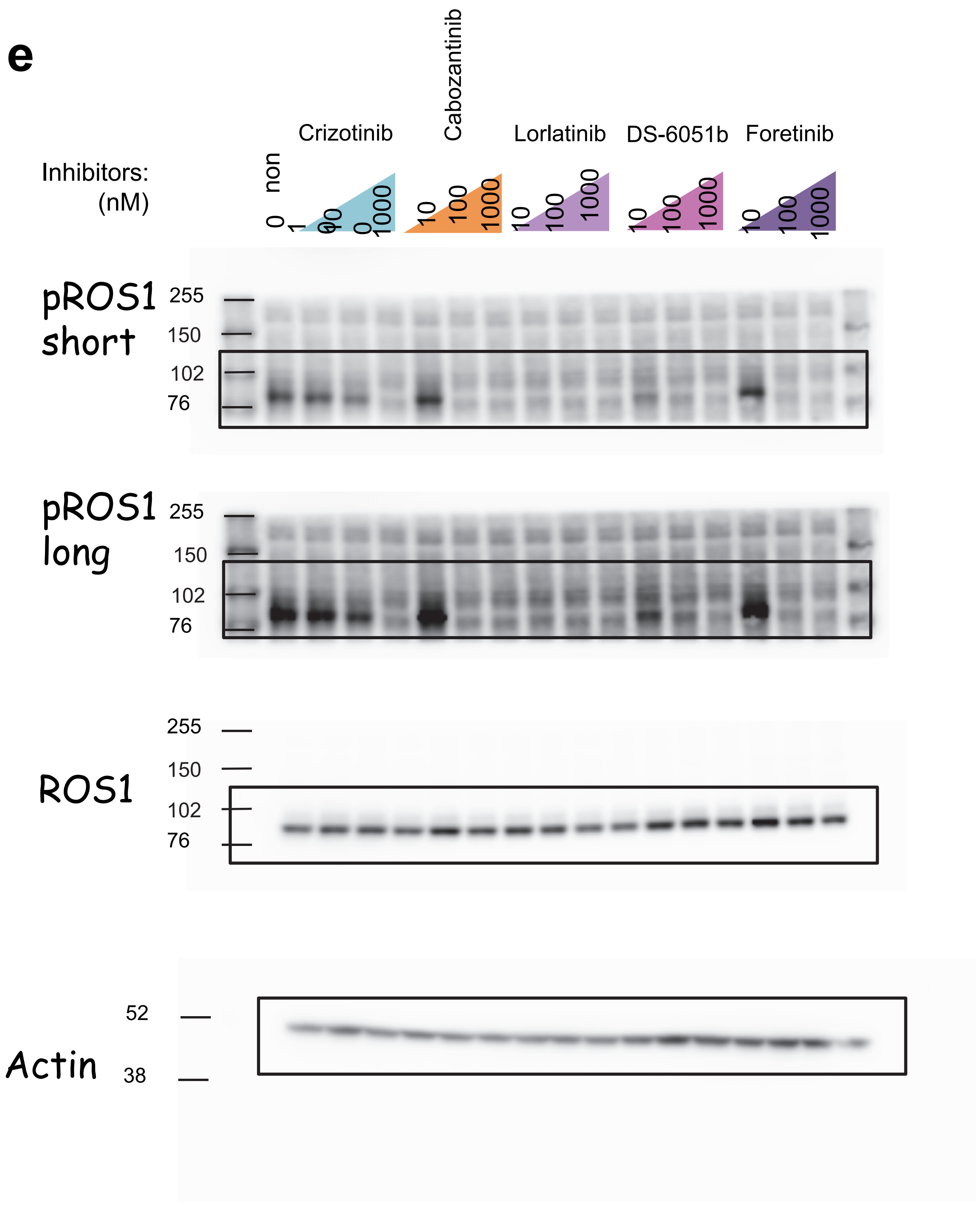

Supplementary Data: Original uncropped image of immunoblots (Fig 2f)

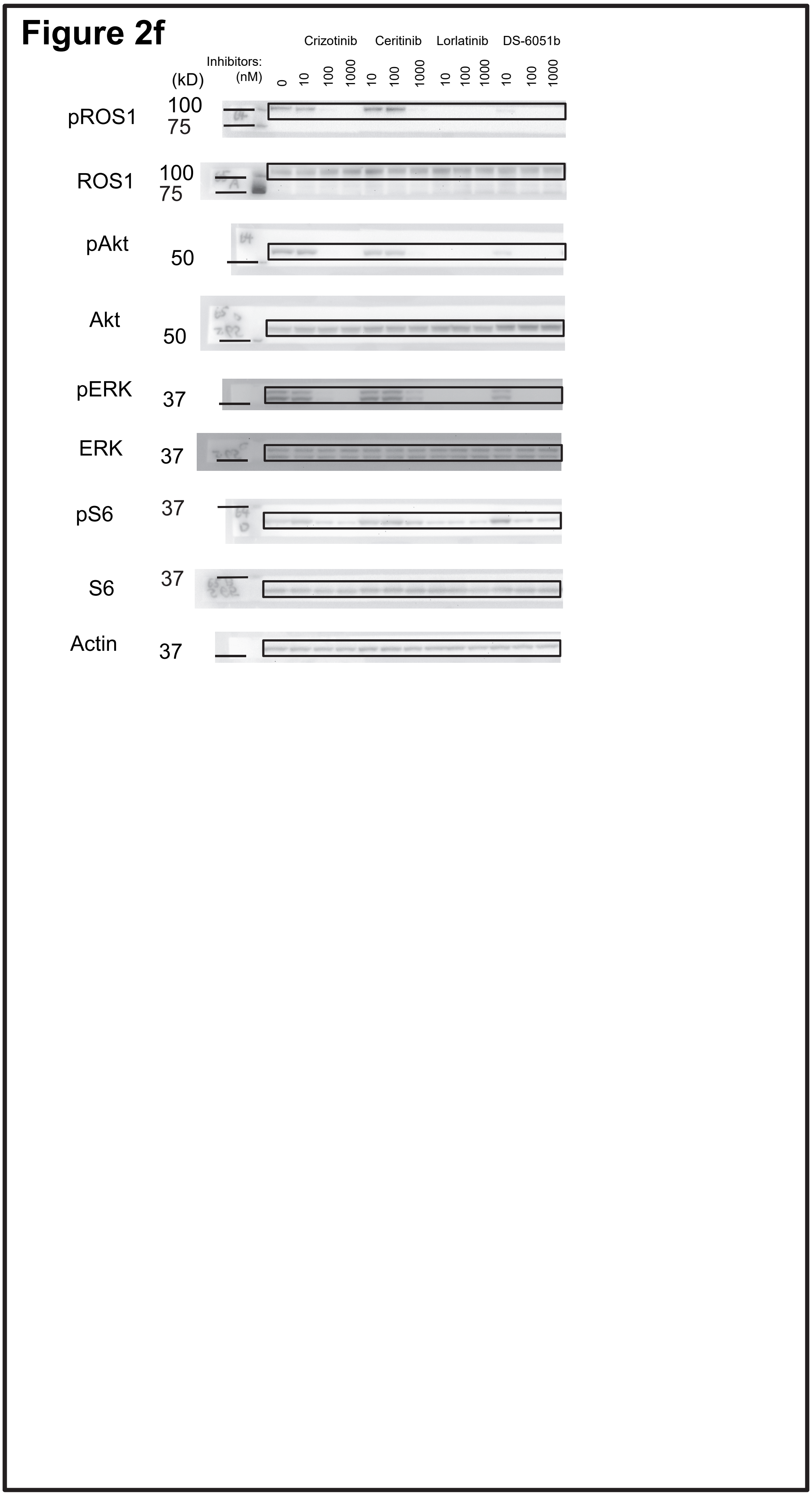

Supplementary Data: Original uncropped image of immunoblots (Fig 2g)

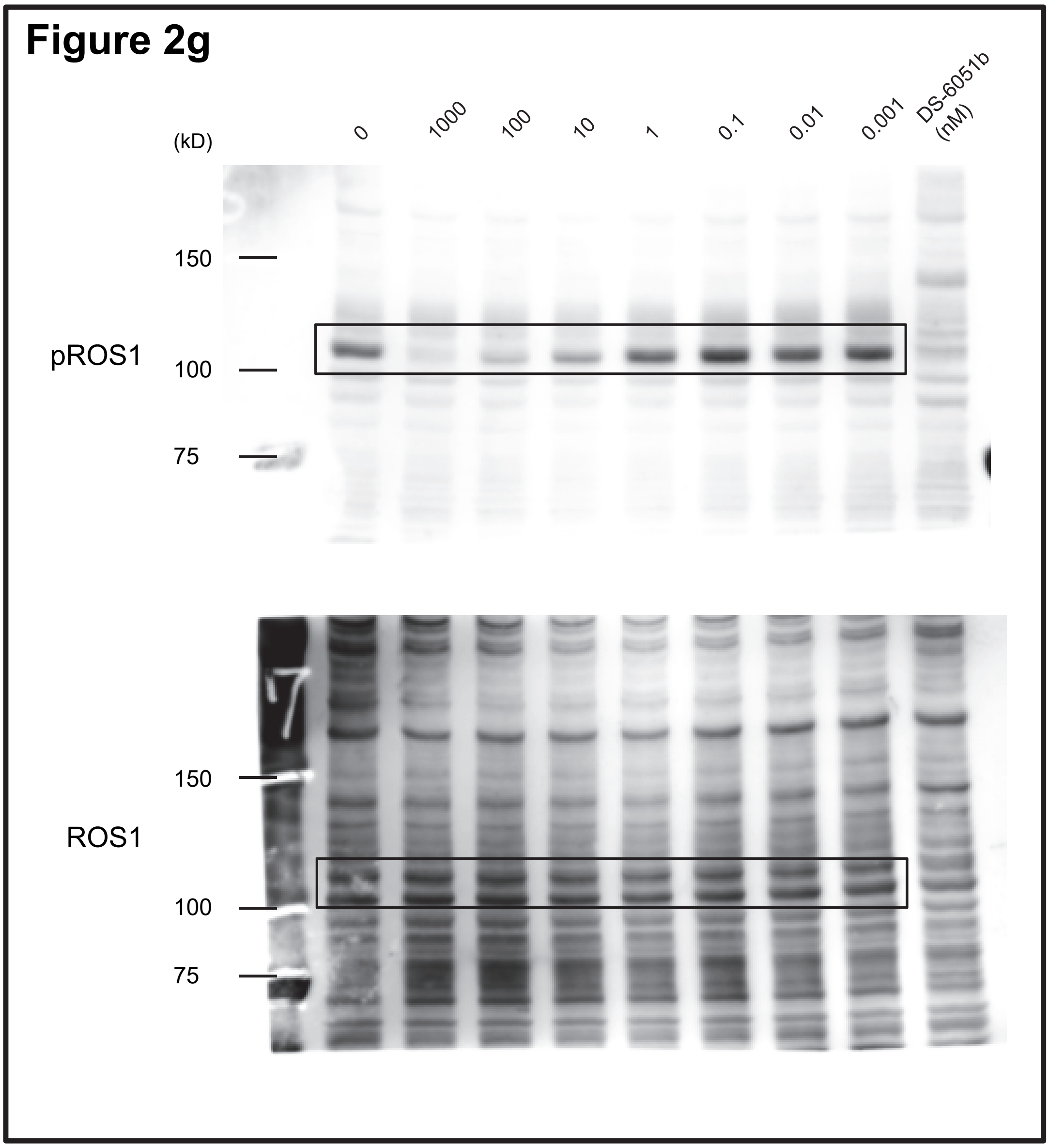

Supplementary Data: Original uncropped image of immunoblots (Fig 3d)

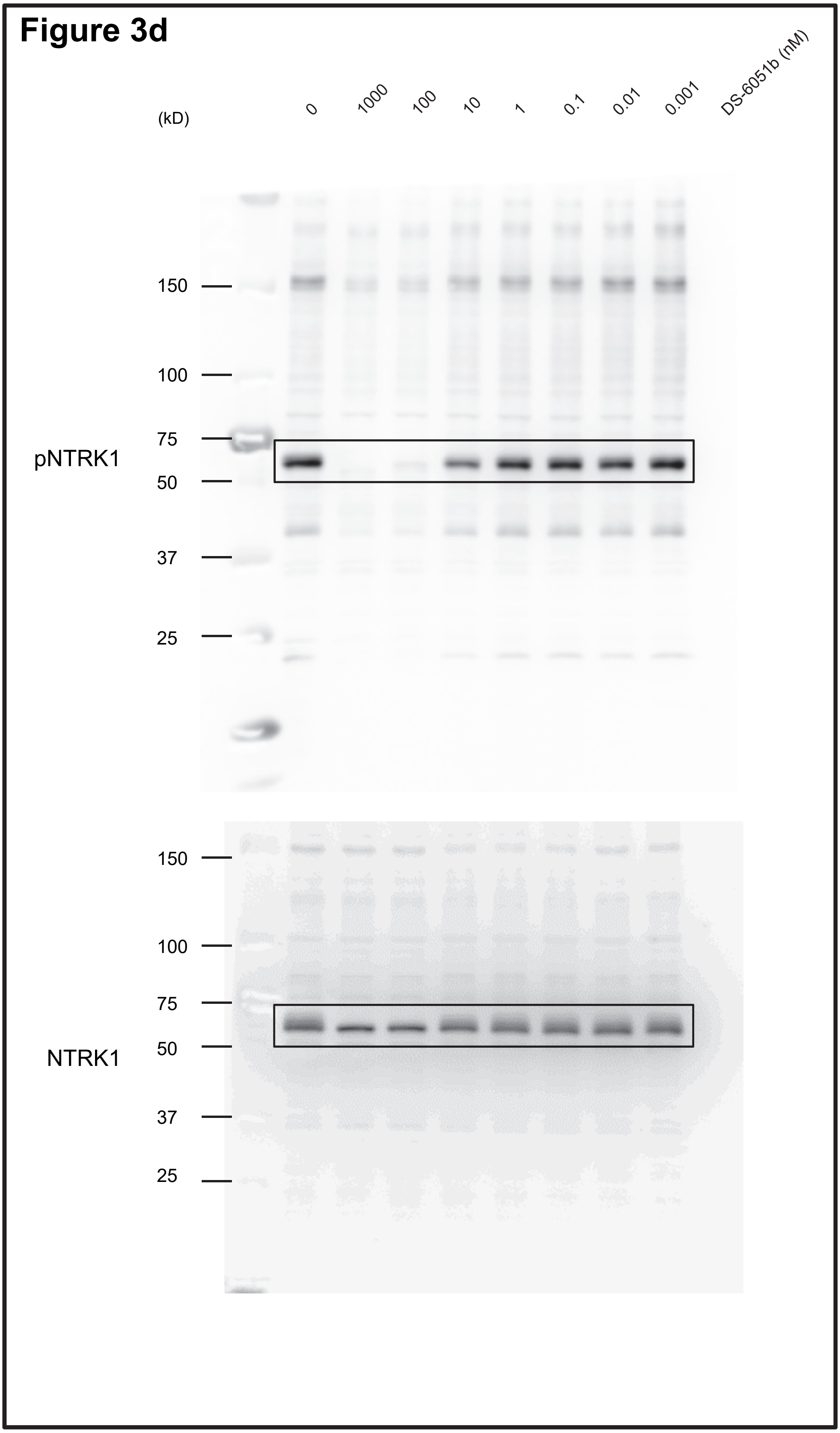

Supplementary Data: Original uncropped image of immunoblots (Fig 4c)

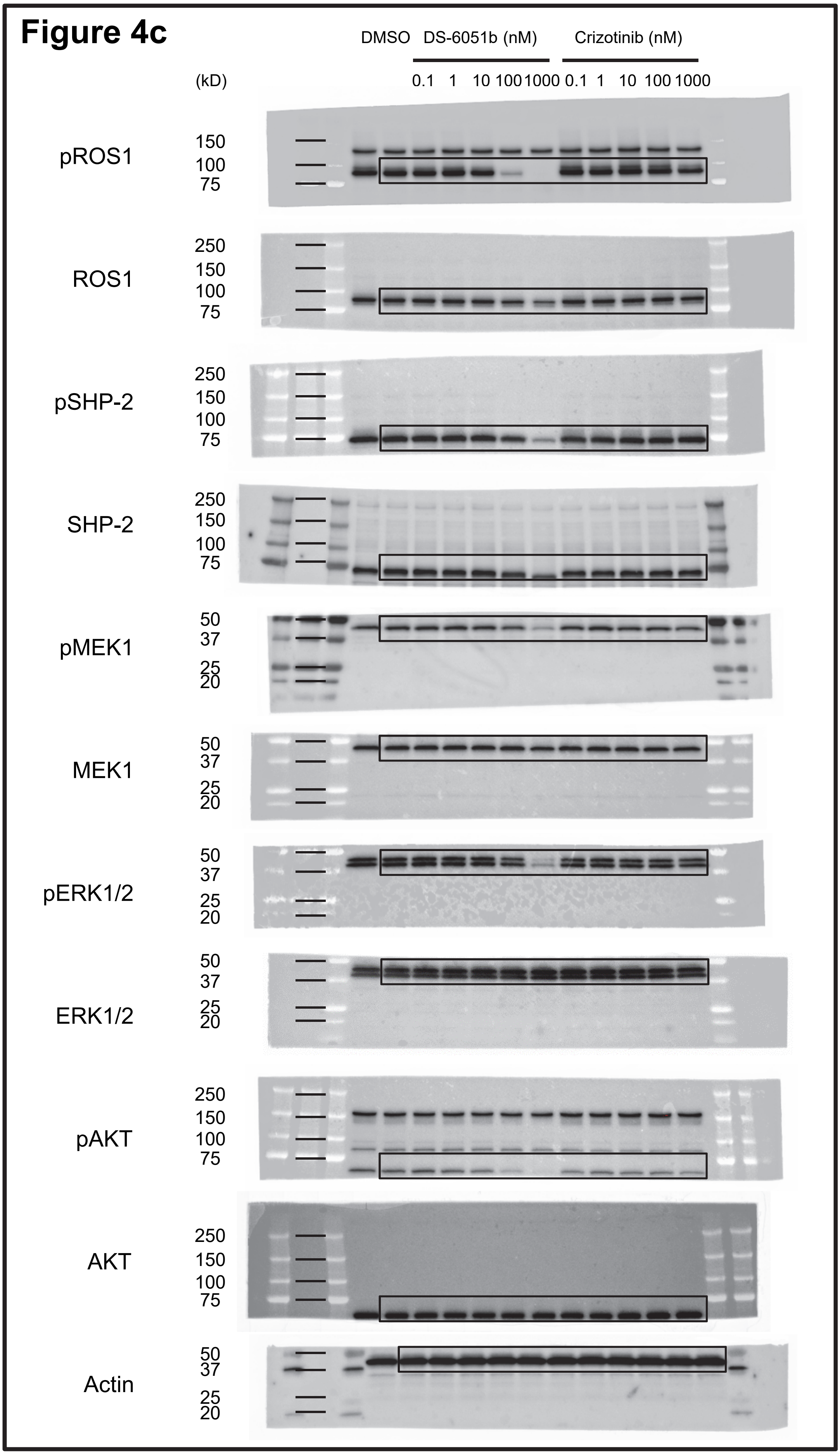

Supplementary Data: Original uncropped image of immunoblots (Fig 5f)

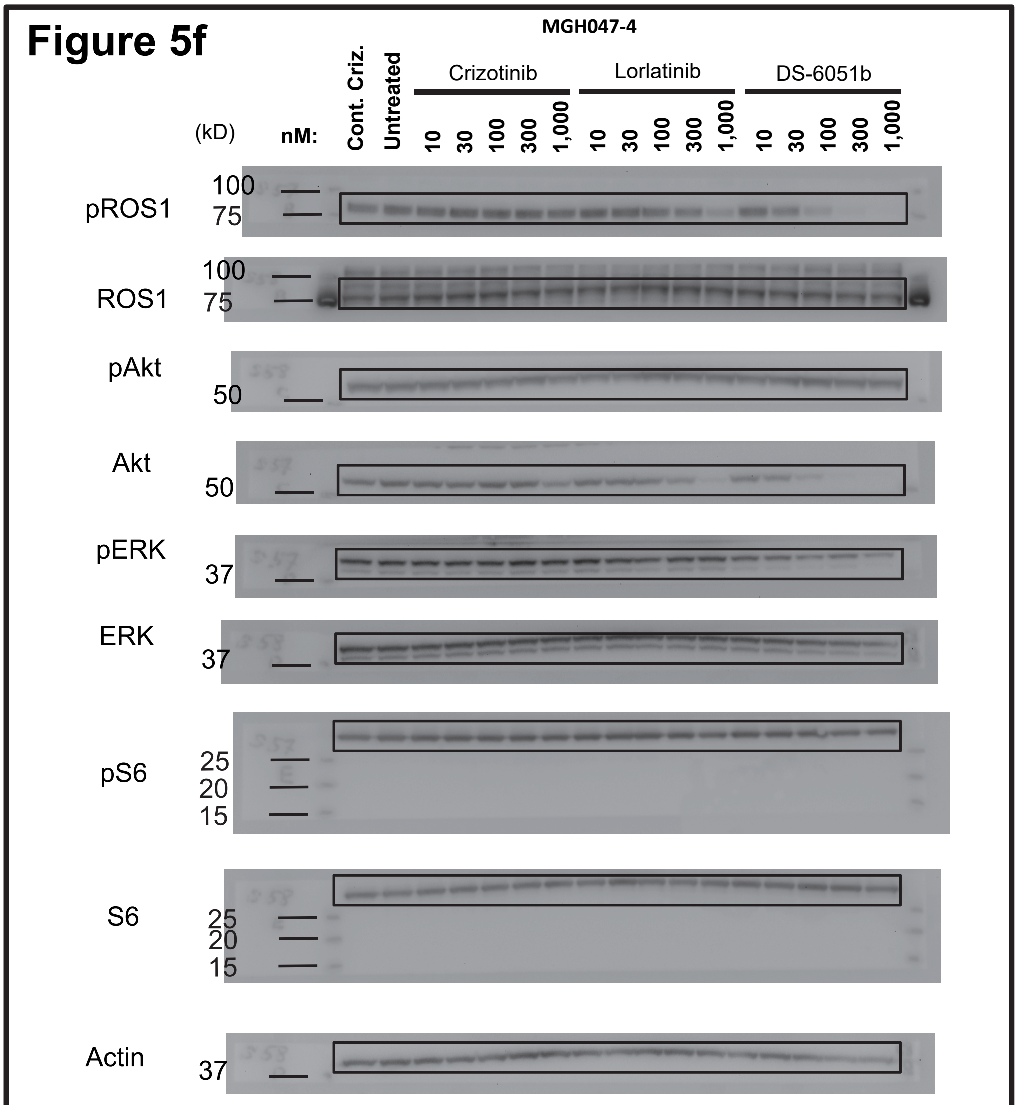

Supplementary Data: Original uncropped image of immunoblots (Fig 6a)

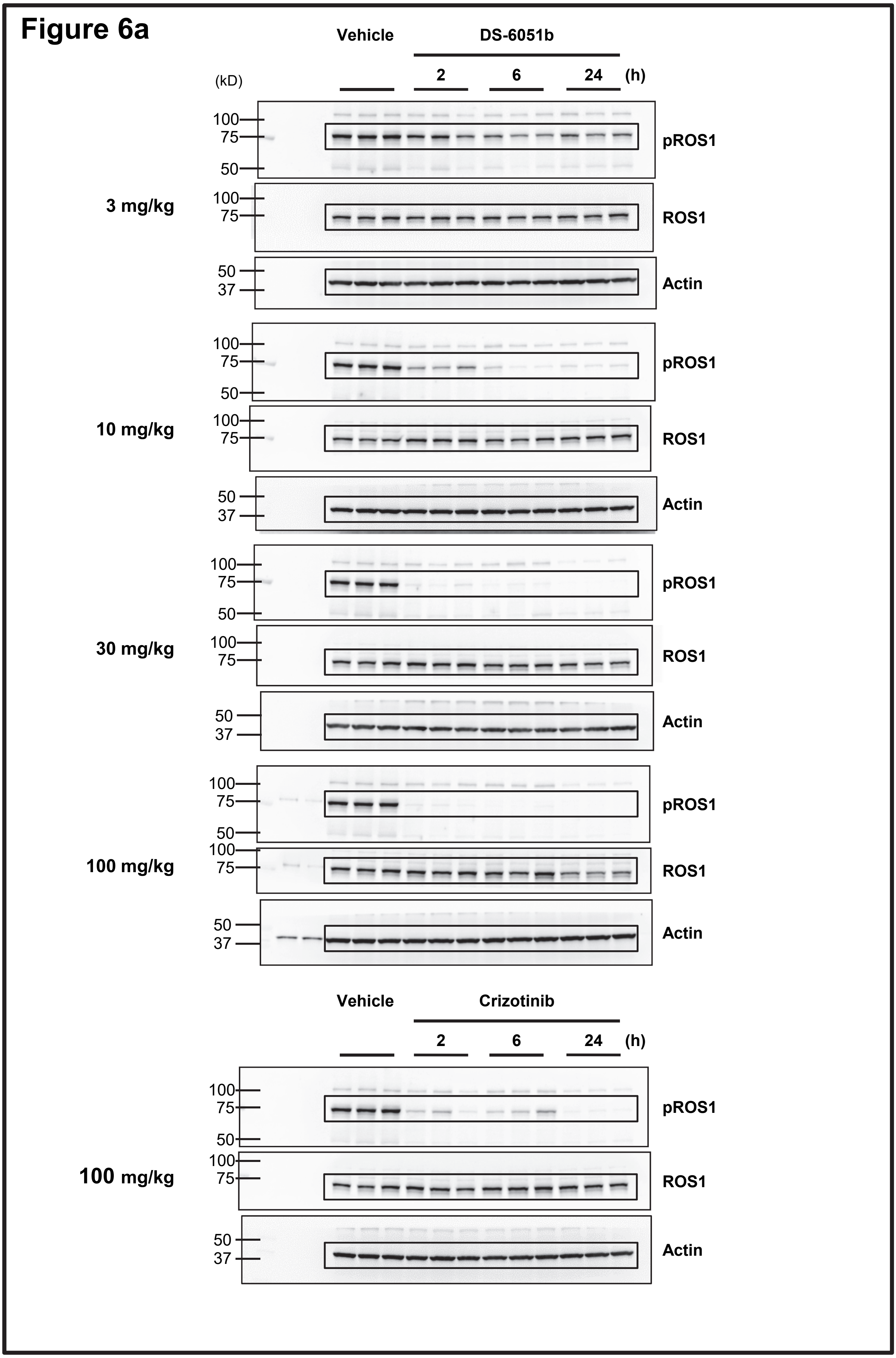

Supplementary Data: Original uncropped image of immunoblots (Fig 6b)

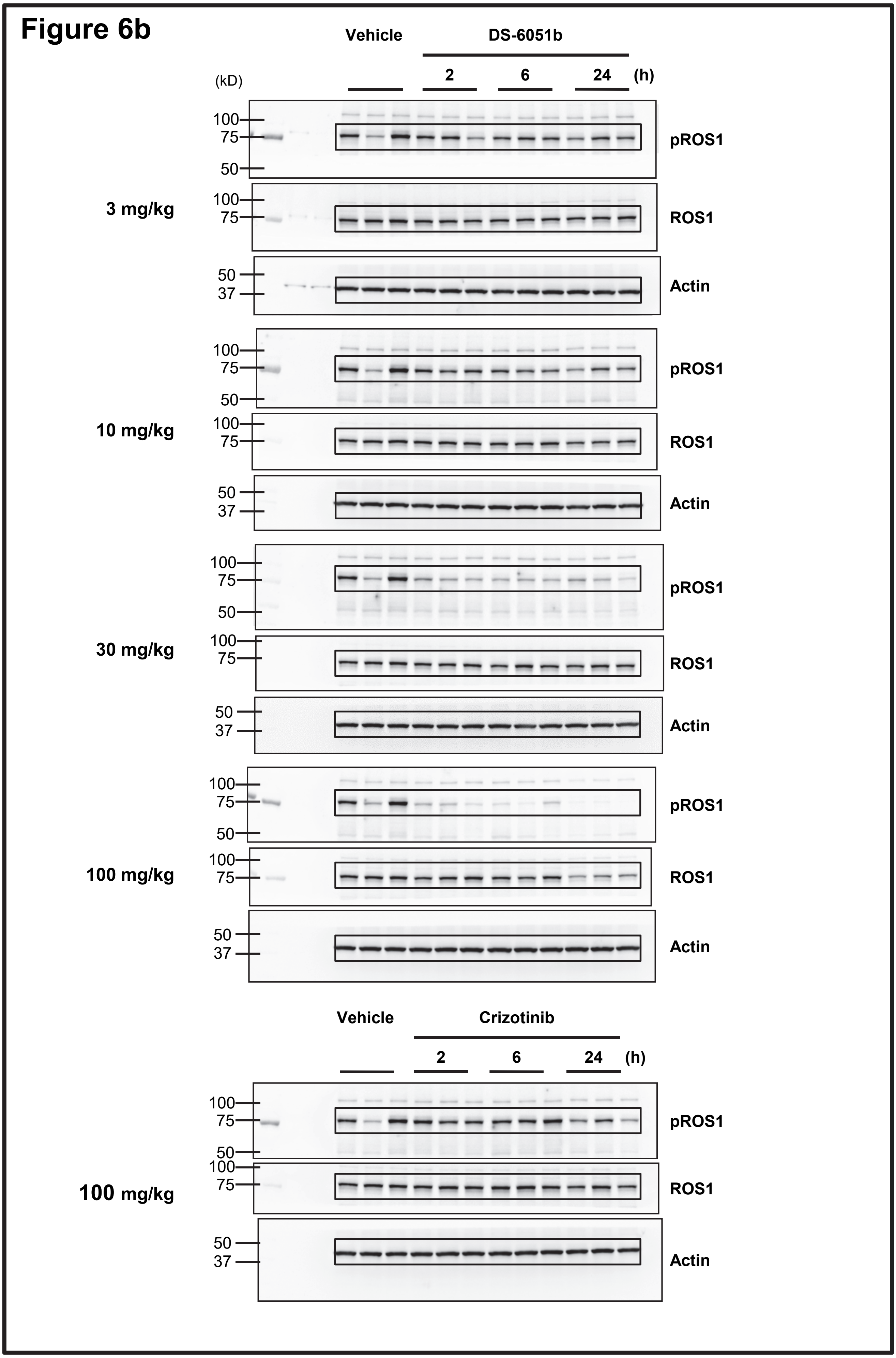

Supplementary Data: Original uncropped image of immunoblots (Fig 6e)

Figure 6e

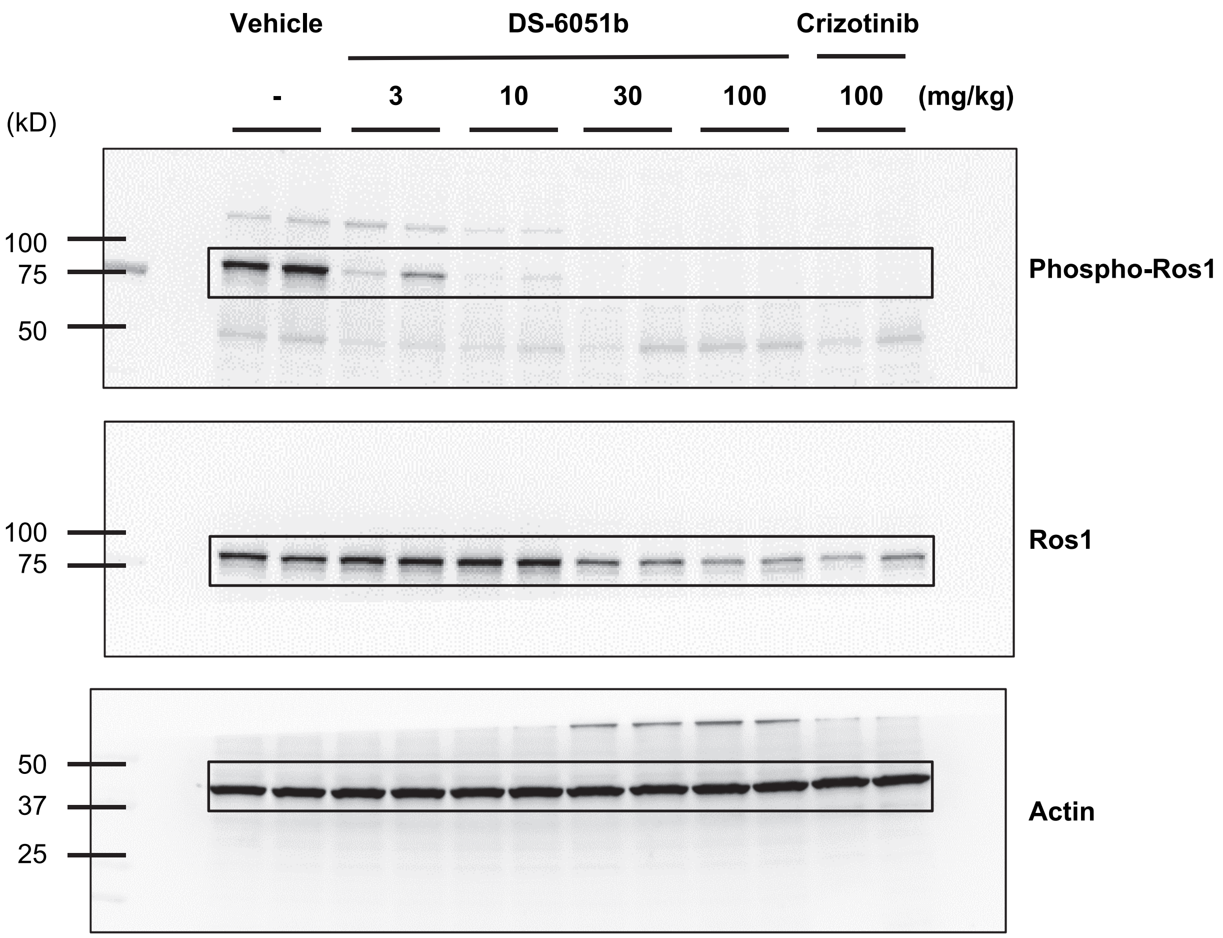

Supplementary Data: Original uncropped image of immunoblots (Fig 6f)

Figure 6f

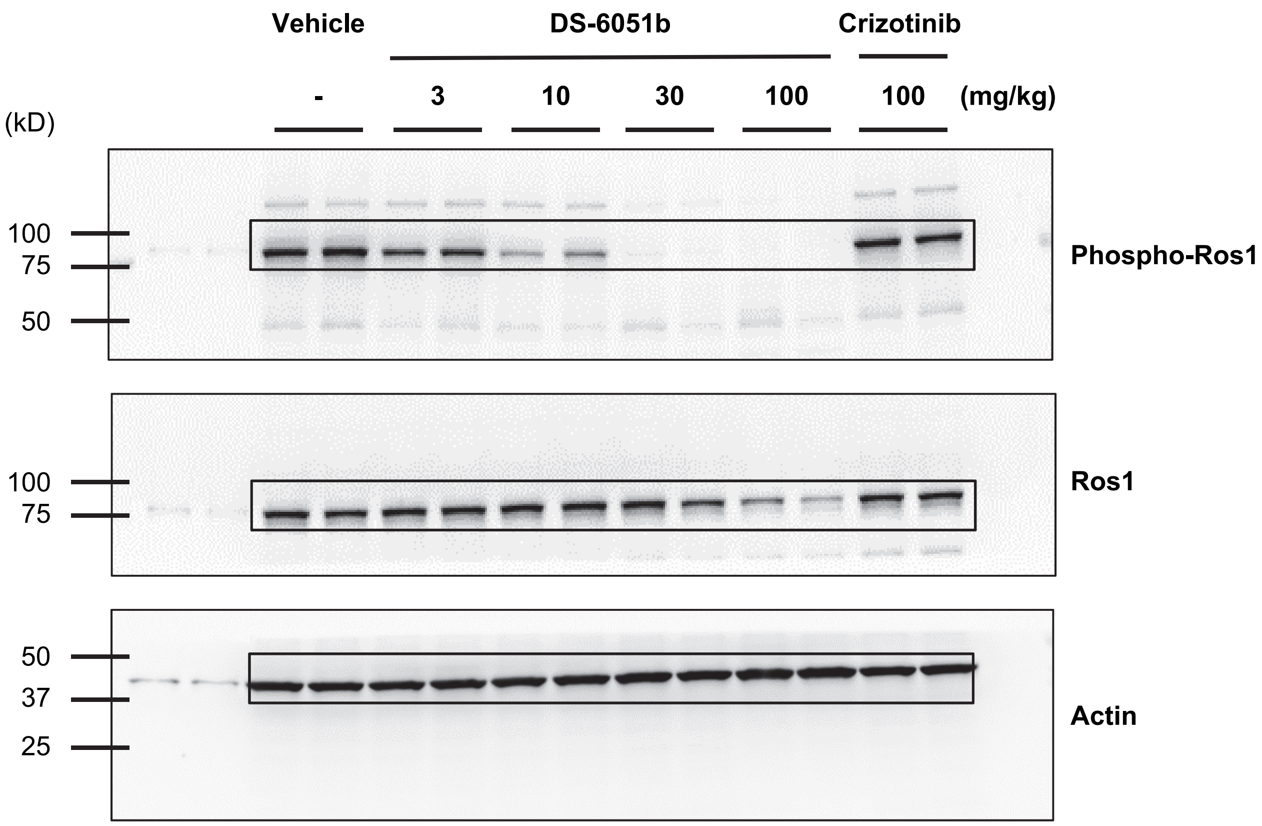

Supplementary Data: Original uncropped image of immunoblots (Fig 7e)

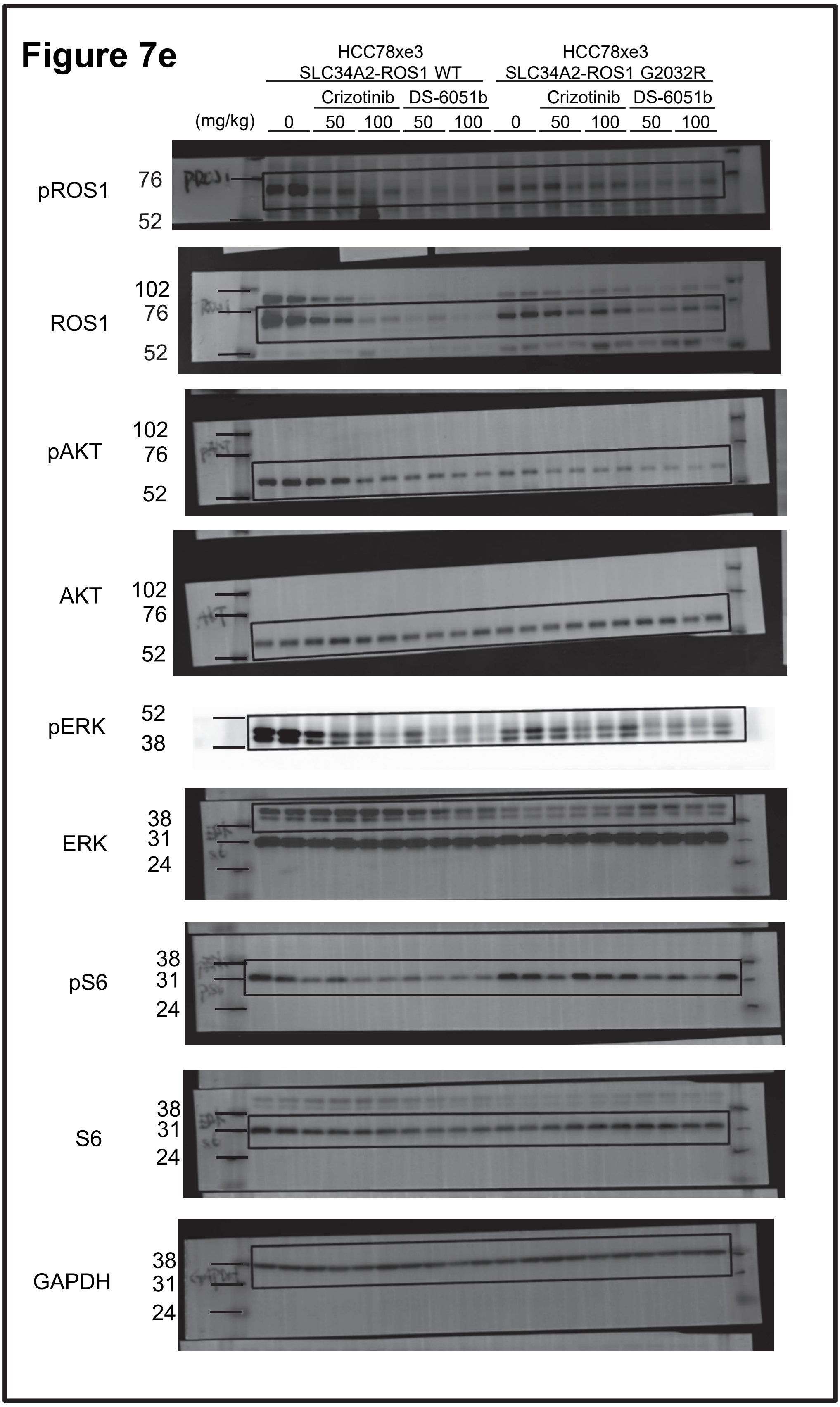

Supplement: Supplementary file 7 — Source Data of Uncropped Immunoblots [file 41467_2019_11496_MOESM7_ESM.pdf]
